# Inhibition of USP1 activates ER stress through Ubi-protein aggregation to induce autophagy and apoptosis in HCC

**DOI:** 10.1038/s41419-022-05341-3

**Published:** 2022-11-10

**Authors:** Longhao Wang, Tao Hu, Zhibo Shen, Yuanyuan Zheng, Qishun Geng, Lifeng Li, Beibei Sha, Miaomiao Li, Yaxin Sun, Yongjun Guo, Wenhua Xue, Dan Xuan, Ping Chen, Jie Zhao

**Affiliations:** 1 Internet Medical and System, Applications of National Engineering Laboratory, The First Afliated Hospital of Zhengzhou University, Zhengzhou, 450052 Henan China; 2 Department of Pharmacy, The First Afliated Hospital of Zhengzhou University, Zhengzhou, 450052 Henan China; 3 Academy of Medical Sciences, School of Basic Medical Sciences, Zhengzhou University, Zhengzhou, 450001 China; 4 The Affiliated Cancer Hospital of Zhengzhou University, Henan Cancer Hospital, No 127, Dongming Road, Zhengzhou, Henan 450008 China; 5 Experimental animal center of Zhengzhou University, Zhengzhou, China

**Keywords:** Chaperone-mediated autophagy, Endoplasmic reticulum

## Abstract

The deubiquitinating enzyme USP1 (ubiquitin-specific protease 1) plays a role in the progression of various tumors, emerging as a potential therapeutic target. This study aimed to determine the role of USP1 as a therapeutic target in hepatocellular carcinoma (HCC). We detected USP1 expression in the tumor and adjacent tissues of patients with HCC using immunohistochemical staining. We evaluated the effect of the USP1 inhibitor ML-323 on HCC cell proliferation and cell cycle using a CCK-8 cell-counting kit and plate cloning assays, and propidium iodide, respectively. Apoptosis was detected by annexin V-FITC/Propidium Iodide (PI) staining and caspase 3 (casp3) activity. Transmission electron microscopy and LC3B immunofluorescence were used to detect autophagy. Western blotting was used to detect the accumulation of ubiquitinated proteins, the expression of endoplasmic reticulum (ER) stress-related proteins, and the AMPK-ULK1/ATG13 signaling pathway. We demonstrated that ML-323 inhibits the growth of HCC cells and induces G1 phase cell cycle arrest by regulating cyclin expression. ML-323 treatment resulted in the accumulation of ubiquitinated proteins, induced ER stress, and triggered Noxa-dependent apoptosis, which was regulated by the Activating Transcription Factor 4(ATF4). Moreover, active ER stress induces protective autophagy by increasing AMPK phosphorylation; therefore, we inhibited ER stress using 4-Phenylbutyric acid (4-PBA), which resulted in ER stress reduction, apoptosis, and autophagy in ML-323-treated HCC cells. In addition, blocking autophagy using the AMPK inhibitor compound C (CC), chloroquine (CQ), or bafilomycin A1 (BafA1) enhanced the cytotoxic effect of ML-323. Our findings revealed that targeting USP1 may be a potential strategy for the treatment of HCC.

## Introduction

Hepatocellular carcinoma (HCC) is one of the leading causes of cancer-related deaths worldwide [1]. Despite great advances in the treatment of liver cancer, the 5-year overall survival (OS) rate of patients with liver cancer remains unsatisfactory. Therefore, further understanding of the molecular and carcinogenic mechanisms of HCC is crucial for identifying new molecular targets for the treatment of liver cancer [2].

Ubiquitination is an important post-translational modification [3]. There is a dynamic equilibrium between ubiquitination and deubiquitination, which maintains the stability of the ubiquitin-protease system [4, 5]. In this system, the deubiquitinating enzyme (DUB) regulates the stability of ubiquitinated proteins by hydrolyzing the ubiquitin chain on the substrate [5–7]. Abnormal DUB activity is associated with many diseases, especially tumor progression [8, 9]. Additionally, ubiquitination and deubiquitination play a vital role in regulating the stability, localization, and metabolism of proteins and controlling the physiological and pathological processes of cells [10, 11]. Ubiquitin-specific peptidase 1 (USP1), which belongs to the DUB family, prevents protein ubiquitination and participates in the regulation of protein stability and degradation [12]. Previous studies have shown that USP1 expression is upregulated in many tumors, exerting a carcinogenic effect. In breast cancer, the upregulation of USP1 expression and deubiquitination of KPNA promotes cell proliferation, migration, and invasion in vitro and promotes lung metastasis of breast cancer cells [13]. In prostate cancer, the stabilization of KDM4A protein by the deubiquitinating enzyme USP1 is a potential treatment strategy [14]. In ovarian cancer cells, targeted USP1 knockout reduces cisplatin resistance cells by regulating the stability of Snail [15]. Moreover, blocking USP1 can inhibit DNA damage repair in osteosarcoma and colorectal cancer cells, trigger tumor cell apoptosis, and enhance the sensitivity to chemotherapeutic drugs [16, 17]. USP1 also plays an important role in leukemia and Fanconi anemia [18–22]. Collectively, these studies demonstrate that USP1 can be used as a potential therapeutic target for cancer; however, its underlying mechanism remains to be further explored.

In recent years, several inhibitors targeting DUBs have been discovered as potential therapeutic strategies [3, 23–26]. ML-323 was first described by Dexheimer et al. as an inhibitor of USP1 and has been since widely used as an anticancer agent in several studies [13, 27–29]. ML-323 treatment inhibits tumor progression in colon cancer, non-small cell lung cancer, breast cancer, prostate cancer, and multiple myeloma [13, 14, 16, 17, 30]. However, the expression and role of USP1 in HCC, as well as the anticancer effect and mechanism of ML-323 on the growth of HCC, remain to be explored.

This study aimed to investigate the expression and role of USP1 in HCC using tumor and adjacent tissues of patients with HCC. We found that USP1 was overexpressed in HCC tissues compared to neighboring tissues. We used USP1 inhibitors as therapeutic reagents in human HCC cell lines, including HCCLM3 and SMMC-7721, to explore their anticancer function and their underlying mechanisms. We also verified the effect of ML-323 on HCC cells in mouse models. We found that ML-323 treatment inhibited the growth of HCC cells by reducing the expression of cyclin D1 and upregulating p27 protein expression levels, leading to cell cycle arrest in the G0/G1 phase. Simultaneously, ML-323 treatment induced the accumulation of ubiquitinated proteins, promoting ER stress and triggering cell apoptosis through the ATF4-Noxa axis. In addition, ER stress promoted an increase in AMPK phosphorylation, which in turn activated protective autophagy. We revealed that the endoplasmic reticulum (ER) stress inhibitors 4-Phenylbutyric acid (4-PBA) and siAMPK inhibited the activation of AMPK and attenuated autophagy in HCC cells. Moreover, USP1 knockout induced G0/G1 cell cycle arrest, apoptosis, and autophagy. Blocking autophagy with the AMPK inhibitors compound C (CC), chloroquine (CQ), or bafilomycin A1 (BafA1) enhanced the cell growth inhibition and apoptosis effects of ML-323 in HCC cell lines. Finally, we found that USP1 inhibition inhibited HCC metastasis. These findings reveal the cytotoxic mechanisms of USP1 inhibitors and suggest potential anticancer strategies for USP1 in HCC.

## Materials and methods

### Cell lines and drug sources

Human HCC cell lines HCCLM3, HepG2, Huh7, and SMMC-7721 were purchased from the Chinese National Infrastructure of Cell Line Resource and cultured in a DMEM medium containing 10% fetal bovine serum (FBS, Gibco, Waltham, MA, USA) at 37 °C and 5% CO2. ML-323, AMPK inhibitor compound C (CC), and 4-phenylbutyric acid (4-PBA) (inhibitor of ER stress) were purchased from Med Chem Express (Shanghai, China) and dissolved in dimethyl sulfoxide (DMSO). Chloroquine (CQ) was purchased from Sigma-Aldrich (St. Louis, MO, USA) and dissolved in phosphate buffer saltwater. Bafilomycin A1 (BafA1) was purchased from Sigma-Aldrich and dissolved in DMSO.

### Immunohistochemical (IHC) staining of human HCC tissue array

The human HCC tissue array was purchased from Yundi Biotechnology (Zhengzhou, China). USP1 expression was detected by IHC staining using a specific USP1 antibody (1:100 dilution, Cat:14346-1-AP, Proteintech Group, Rosemont, IL, USA). Briefly, the tissue array section (4 μm) was dehydrated, and peroxidase was blocked. A 0.01 mol/L citric acid buffer (pH 6.0) and a pressure cooker were used for antigen recovery. Tissues were then incubated with primary antibodies overnight at 4 °C, then stained with tissue staining kits (SP-9000) and 3,3′-diaminobenzidine tetrahydrochloride (DAB) (ZLI-9032; ZSGB-BIO, Beijing, China). The slides were counterstained with hematoxylin and eosin (HE). The stained glass slides were observed and an image was obtained through a microscope. Based on the staining intensity, the samples were divided into four groups, from the lowest intensity (−) to the highest intensity (+++) groups.

### Cell viability and clonogenic survival assay

HCC cell lines HCCLM3, HepG2, Huh7, and SMMC-7721 were uniformly seeded in a 96-well plate (3 × 10^3^ cells/well) and treated with ML-323 or DMSO (0.1%) for 48 h. Cell viability was determined using a cell-counting-kit (CCK)-8 (Beyotime Biotechnology, Nantong, China) according to the manufacturer’s protocol. For the colony formation assay, 500 cells were seeded into a 6-well plate in triplicate, treated with DMSO or ML-323, and incubated for 10 d. The colonies were treated with 4% paraformaldehyde (Solarbio, Beijing, China) and crystal violet (Solarbio Science & Technology Co., Ltd, Beijing, China), then counted.

### Cell cycle analysis

HCCLM3, HepG2, Huh7, and SMMC-7721 cells were treated with DMSO or ML-323 for 24 h. The cells were collected and fixed with 70% absolute ethanol at 4 °C overnight, and then propidium iodide solution (50 μg/mL; Solarbio) containing RNase A (30 μg/ml; Solarbio) was added at 37 °C for 30 min and detected by flow cytometry (NOVOCYte3130; ACEA Biosciences, Hangzhou, China).

### Detection of apoptosis and caspase3 activity

HCCLM3 and SMMC-7721 cells were treated with DMSO or ML-323 at specific concentrations for 48 h. The AnnexinV-EGFP/PI double-stained cell apoptosis detection kit (KeyGEN BioTECH, Nanjing, China) was used according to the manufacturer’s instructions. The activity of caspase 3 (CASP3) was measured using a Fluorescein Active Caspase3 staining kit (BioVision Inc., Milpitas, CA, USA) according to the manufacturer’s instructions.

### Evaluation of mitochondrial membrane depolarization

HCCLM3 and SMMC-7721 cells were treated with DMSO or ML-323 for 24 h. The cells were collected and tested for mitochondrial membrane depolarization using a mitochondrial membrane potential measurement kit containing JC-1 according to the manufacturer’s protocol (Yeasen Biotechnology, Shanghai, China). Data were obtained and analyzed using flow cytometry. Cells with intact mitochondria showed high red fluorescence and appear in the upper-right quadrant of the scatter plot. In contrast, cells that have lost mitochondrial membrane potential appear in the lower right quadrant, showing prominent green and low red fluorescence.

### Western blotting assay

HCCLM3 and SMMC-7721 cells were treated with DMSO or ML-323 for 48 h, collected, and lysed with lysis buffer RIPA (Beyotime) for western blot analysis. Antibodies against p27(#3686), CyclinD1(#55506), CyclinE1(#4129), CDK2(#18048), CDK4(#23972), ATF4(#11815), LC3B (#3868), AMPKɑ (#5832), ATG13(#13273), Phospho-ULK1(#14202), Phospho-eif2α(Ser51) (#3398), eif2α(#5324), FOXO3a(#12829), cleaved CASP9(#7237), cleaved CASP3(#9664), cleaved-PARP(#5625), Bax(##5023), Bak(#12105), Bcl-xl(#2764), Mcl-1(#5453), XIAP(#2045), CIAP1(#7065), Bcl-2(#15071), Noxa(#14766), and c-Myc(#18583) were obtained from CST (Cell Signaling Technology, Danvers, MA, USA). Antibodies against Ubiquitin (sc-8017) was obtained from Santa (Santa Cruz Biotechnology, Dallas, Tx, USA). Antibodies against ATG5(AF2269), and Phospho-AMPKɑ (Thr172) (AA393) were obtained from Beyotime (Beyotime, Shanghai, China). Antibodies against USP1(Cat No. 14346-1-AP) was obtained from Proteintech (Proteintech, Wuhan, China). Antibodies against GAPDH (TA-08, ZGSB-Bio, China), α-tubulin (TA-10, ZGSB-Bio, China), and β-actin (TA-09, ZGSB-Bio, China) were used as loading controls. All primary antibodies were diluted at 1:1000 and incubated at 4 °C overnight. The secondary antibodies, peroxidase-conjugated goat anti-mouse IgG, and peroxidase-conjugated goat anti-rabbit IgG were purchased from ZGSB-Bio, Inc. (Beijing, China). Membranes (PVDF) were incubated with the corresponding secondary antibody (dilution: 1:3000) for 2 h at 25 °C and detected using the ECL kit (Beyotime).

### Gene silencing using siRNA

HCCLM3 and SMMC-7721 cells were transfected with synthetic siRNA oligonucleotides (final concentration:100 nmol/L). Lipofectamine 8000 (Beyotime) was purchased from Gene Pharma (Shanghai, China). The siRNA sequences were as follows: siNoxa: GUAAUUAUUGACACAUUUC; ^31^siATF4:GCCUAGGUCUCUAGAUGA [31]; siControl: GUUCUCCGAACGUGUCACGU; siUSP1-1:UCUCCGAACGUGUCACGU [32]; siUSP1-2:GGUUAAAGUCUGCAACUAATT; siAMPK: CAAUAAGGCUCAUGCACAA; siATG5: CCTGAACAGAATCATCCTTAA.

### Immunofluorescence staining

HCCLM3 and SMMC-7721 cells were placed in a glass-bottom cell culture dish and treated with 0.1% DMSO or ML-323 (80 μmol/L) for 24 h. The cells were fixed with anhydrous methanol at −20 °C for 25–30 min, blocked with 5% bovine serum albumin, and then with LC3B primary antibody (1:300, overnight at 4 °C; Cell Signaling Technology) and AlexaFluor488® goat anti-rabbit IgG (H+L) secondary antibody (green) (1:500, 2 h at 25 °C in the dark; Beyotime) were incubated separately. The nuclei were stained with DAPI blue (5 μg/ml, 20 min at 25 °C, in the dark; Beyotime). A fluorescence microscope (magnification:200×; OLYMPUS, OLYMPUS Corporation, Japan) was used.

### Transmission electron microscopy

SMMC-7721 cells were treated with 0.1% (DMSO or ML-323 (80 μM) for 24 h, fixed with 2.5% glutaraldehyde solution (G1102, servicebio technology CO., LTD, Wuhan) for 2–4 h at 4 °C, and then fixed with 1% osmic acid·0.1 M phosphate buffer PB (PH7.4) at 25 °C for 2 h. The dehydrated cells were embedded and sliced (60–80 nm), double-stained with uranium and lead, a representative area was selected for observation under a transmission electron microscope, and image analysis was performed (HT7700, HITACHI).

### Mouse model of human HCC and treatment

We chose 5-week-old female BALB/c nude mice (the animals in the study were purchased from Beijing Vital River Laboratory Animal Technology Co., Ltd.) for tumor xenografts experiments. BALB/c nude mice were injected subcutaneously with 2 × 10^6^ HCCLM3 cells [33, 34]. The tumor-bearing mice were randomly divided into four groups, each group included five mice (*n* = 5) and treated with 10% 2-hydroxypropyl-β-cyclodextrin (HPBCD) (Sigma-Aldrich) or ML-323 (20 mg/kg) or sorafenib (30 mg/kg) or ML-323 sorafenib every 3 days. Tumor growth was observed, the weight change of the mice was recorded, and tumor size was determined by caliper measurements. The ellipsoid volume formula (length × width 2/2) was used to calculate tumor volume. Mice were photographed and weighed upon sacrifice. All mice were euthanized, and the tumors were surgically removed. The tumor tissue was collected and fixed in liquid nitrogen or 4% paraformaldehyde for subsequent analysis. Investigators were blinded to the treatment groups during data collection and subsequent data analysis. All experiments on mice were carried out in the SPF-grade animal feeding room with adequate food and water. Animal experiments were performed in accordance with the animal experimental protocol approved by the Institutional Animal Care and Use Committee of Zhengzhou University.

### Statistical analysis

SPSS21.0 and GraphPad Prism 8 software were used for statistical analysis of experimental data. Analysis of variance (ANOVA) and Student’s *t*-test were used for those that conformed to the normal distribution. The Kaplan–Meier method was used for survival analysis. The *t*-test was used to compare parameters between the groups. The Mann–Whitney *U*-test was applied in comparison of tumor volume. Categorical data were evaluated with the χ2 test or Fisher’s exact test. For all tests, * indicates that the difference between the two groups was significant (*P < 0.05, **P < 0.01, ***P < 0.001).

## Results

### USP1 is overexpressed in human HCC tissues

**Fig. 1:**
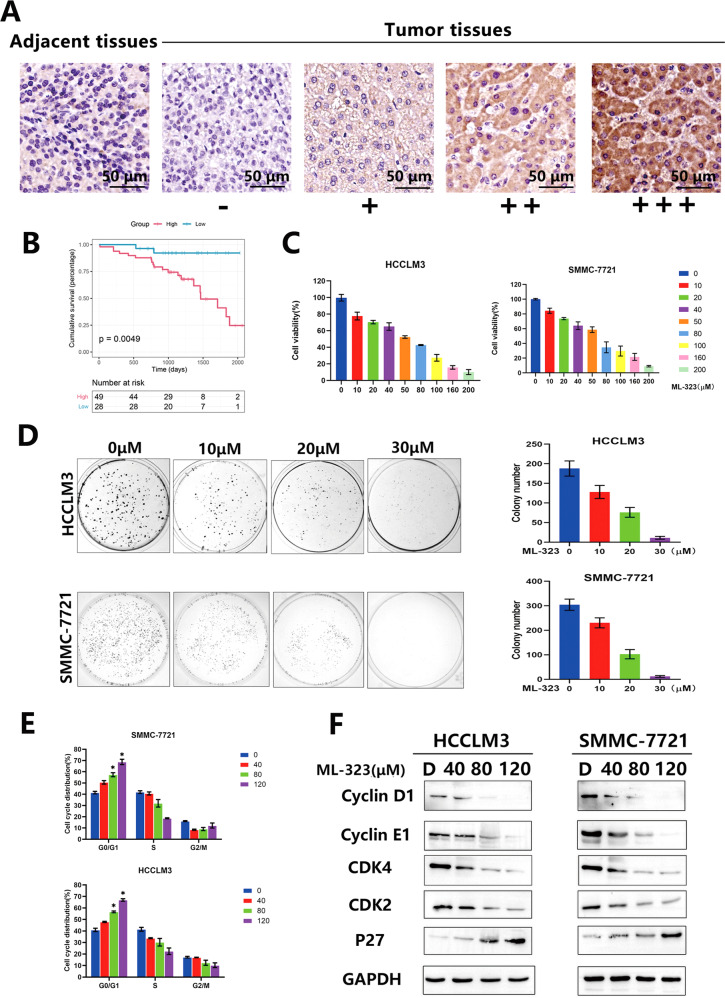
USP1 is overexpressed in hepatocarcinoma cancer (HCC) tissues. The USP1 inhibitor ML-323 inhibits HCC cell proliferation and induces cell cycle arrest. **A** Immunohistochemical staining of USP1 in human HCC tissue arrays. Samples were divided into four groups according to the staining intensity, from the weakest (Group 1) to the strongest (+++, Group 4). **B** The Kaplan–Meier curve of the overall survival rate of patients with HCC was obtained based on USP1 expression (*P* = 0.0049, log-rank test). Groups 1–2 were low-expression groups and groups 3–4 were high-expression groups. **C** Effects of ML-323 on the activities of HCCLM3 and SMMC-7721 cells. The cells were treated with 0.1% DMSO or ML-323 for 48 h, and cell viability was determined using a cell-counting kit (CCK)-8 kit. **D** Effect of ML-323 on HCC cell colony formation. HCC cells were treated with different concentrations of ML-323 for 10 d and then fixed, stained, and counted. Colony collection is shown on the left, and the colony number for statistical analysis is shown on the right.ML-323 induces G0/G1 cell cycle arrest in HCC cells. HCCLM3 and smmc-7721 cells were treated with different concentrations of ML-323 for 24 h, GraphPad software for distribution analysis. **E** ML-323 induced G0/G1 cell cycle arrest in HCC cells. HCCLM3 and SMMC-7721 cells were treated with different concentrations of ML-323 for 24 h. **F** Western blot analysis showing the effects of ML-323 on the expression of cell cycle-related proteins. HCCLM3 and SMMC-7721 cells were treated with ML-323 at the specified concentrations. GAPDH was used as a loading control. Data represent at least three independent experiments (*n* = 3; error bars, SD). GAPDH was used as the loading control. All data were representative of at least three independent experiments (*n* = 3; error bar, SD).

To investigate the clinical significance of USP1 in HCC, the expression of USP1 was first detected by IHC staining of human HCC tissue arrays. The samples were divided into four groups, with staining intensities ranging from weakest (−) to strongest (+++) (Fig. 1A). In addition, Kaplan–Meier analysis showed that increased USP1 levels were negatively correlated with the 5-year overall survival (OS) rate of patients with HCC (*P* = 0.0049, log-rank test; Fig. 1B). These results indicated that USP1 is overexpressed in HCC cells and is negatively correlated with the OS.

### The USP1 inhibitor ML-323 suppresses HCC cell growth

To determine whether USP1 can be used as an anti-HCC target, the anticancer effect of the USP1 inhibitor ML-323 was studied by treating various HCC cell lines with different ML-323 concentrations and evaluating cell viability. The results showed that ML-323 (0–200 μM) inhibited the growth of HCCLM3 and SMMC-7721 HCC cells in a dose-dependent manner (Fig. 1C, and Supplementary Fig. 1A) and inhibited colony formation (Fig. 1D and Supplementary Fig. 1B). These results indicated that targeting USP1 is a potential therapeutic strategy to treat HCC.

### ML-323 induced G1 phase cell cycle arrest in liver cancer cells

To clarify how ML-323 inhibits HCC cell growth, we first studied the effects of ML-323 on the cell cycle using flow cytometry analysis. The results showed that after 24 h of ML-323 treatment, the cell cycle was arrested in the G0/G1 phase (Fig. 1E and Supplementary Fig. 1C, D). The expression of cell cycle-related proteins was detected by western blot analysis. The results showed that ML-323 treatment increased G0/G1 phase-related protein p27 and decreased the expression of CyclinD1, CylinE1, CDK2, and CDK4 (Fig. 1F and Supplementary Fig. 2D). This result indicated that ML-323-treated cells stagnated during mitosis and were arrested in the G1 phase.

### Noxa plays a vital role in ML-323-induced intrinsic apoptosis

**Fig. 2:**
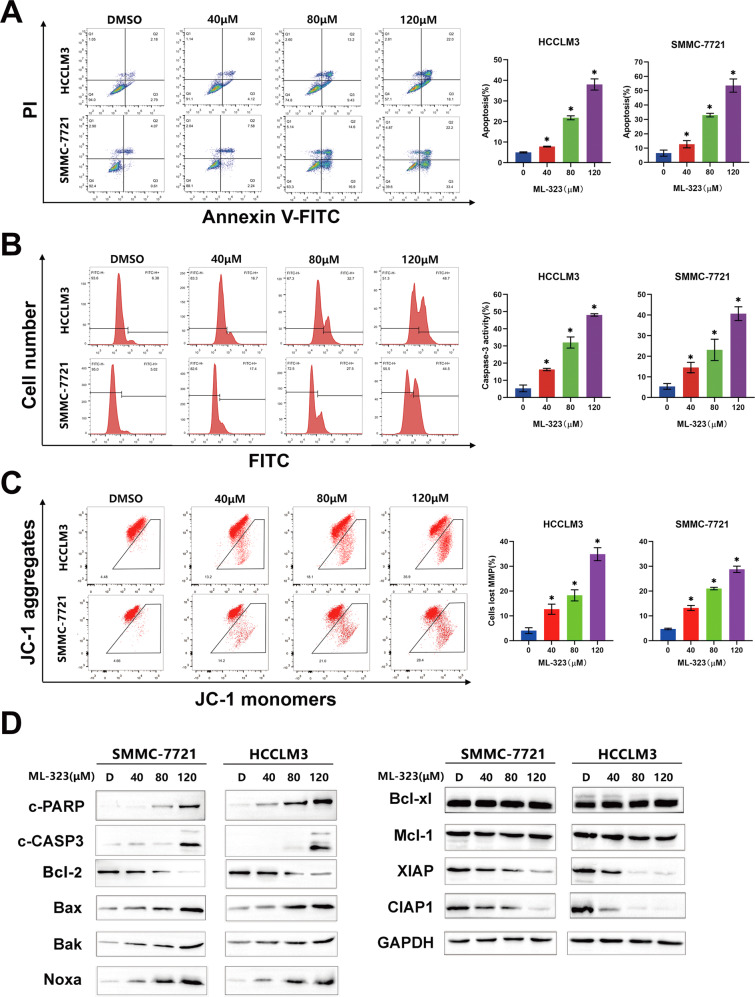
ML-323 induces intrinsic apoptosis in hepatocarcinoma cancer (HCC) cells. **A** Fluorescence-activated cell sorting (FACS) analysis using an Annexin V-FITC/PI double-staining kit. Annexin V+ cell populations were defined as apoptotic cells. **B** FACS showing CASP3 activity in ML-323-treated HCC cells. Data are representative of at least three independent experiments. **C** FACS analysis of mitochondrial membrane depolarization after ML-323 treatment. **D** Western blot analysis showing the effect of ML-323 on the expression of apoptotic, pro-apoptotic, and anti-apoptotic proteins. HCCLM3 and SMMC-7721 cells were treated with ML-323 for 48 h. GAPDH was used as a loading control. All the data represent at least three independent experiments (*n* = 3; error bars, SD).

We investigated whether ML-323 exerts its growth inhibitory effect via apoptosis using the annexin V apoptotic assay and by evaluating apoptotic markers. The results showed that ML-323 treatment induced apoptosis and significantly increased the number of annexin V cells (Fig. 2A and Supplementary Fig. 2A, B) and caspase-3 (CASP3)-activated cells (Fig. 2B). In addition, ML-323 effectively induced the lysis of CASP3 and PARP (Fig. 2D and Supplementary Fig. 2D). These results suggested that ML-323 triggers apoptosis in HCC cells.

**Fig. 3:**
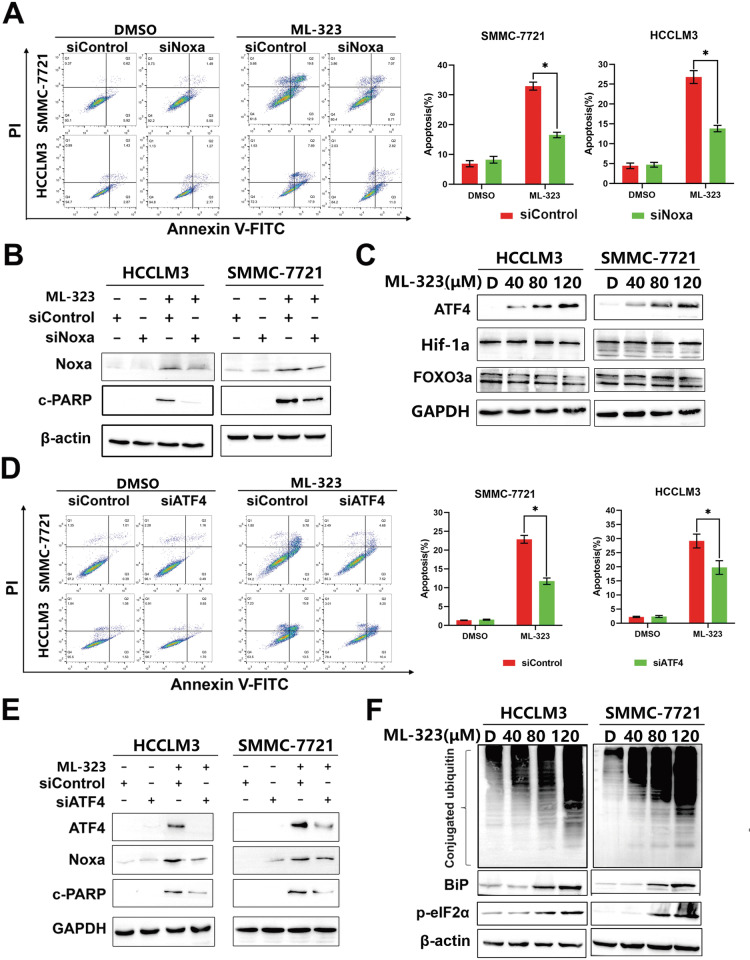
ML-323 induces hepatocarcinoma cancer (HCC) cell apoptosis via the ATF4-Noxa axis. **A** Annexin V-FITC /PI double-staining showing apoptotic Noxa-deficient HCC cells treated with ML-323. HCCLM3 and SMMC-7721 cells were transfected with control siRNA or Noxa siRNA and then treated with ML-323 for 48 h. **B** Western blot analysis showing the efficiency of siNoxa and its effects on cleaved-PARP levels. HCCLM3 and SMMC-7721 cells were treated as shown in (**A**). **C** Western blot analysis of Noxa-related transcription factors. HCCLM3 and SMMC-7721 cells were treated with DMSO or ML-323, and cell lysates were analyzed by western blotting using specific antibodies. **D** Annexin V-FITC /PI double-staining of ATF4-deficient HCC cells treated with ML-323. After transfection with control or ATF4 siRNA, HCCLM3 and SMMC-7721 cells were treated with ML-323 for 48 h. **E** Western blot analysis of the knockdown efficiency of siATF4 and its effect on the expression of Noxa and cleaved-PARP. **F** Western blot analysis of ubiquitinated proteins and ER stress-related proteins. HCCLM3 and SMMC-7721 cells were treated with ML-323. All the data represent at least three independent experiments (*n* = 3; error bars, SD). All data represented at least three independent experiments (*n* = 3; Error bars, SD).

We also detected the changes in the mitochondrial membrane potential, which is another classical sign of intrinsic apoptosis activation. The results showed that ML-323 treatment resulted in the loss of matrix metalloproteinases (Fig. 2C), further confirming the induction of intrinsic apoptosis. Moreover, ML-323-treated HCCLM3 and SMMC-7721 cells expressed BCL-2 and its family members, including pro-apoptotic (Noxa, Bak, and Bax) and anti-apoptotic proteins (Bcl-xl, Mcl-1, CIAP1, and XIAP). Among these proteins, the pro-apoptotic protein Noxa was significantly upregulated in these treated cells compared to the non-treated cells (Fig. 2D and Supplementary Fig. 2D). In addition, the downregulation of Noxa by siRNA silencing markedly inhibited ML-323-induced apoptosis and reduced the number of annexin V cells (Fig. 3A) and PARP cleavage (Fig. 3B). These findings emphasized the key role of Noxa in ML-323-induced intrinsic apoptosis in HCC cells.

### ML-323 triggers ER stress and activates the ATF4-Noxa Axis to induce intrinsic apoptosis

Noxa is a key mediator of apoptosis and is transactivated by various transcription factors35. Therefore, we evaluated the changes in the expression of Noxa-regulated transcription factors after ML-323 treatment. The results showed that ML-323 treatment increased the protein levels of activated transcription factor 4 (ATF4) and only slightly affected the expression of other transcription factors (Fig. 3C). In addition, ATF4 knockout rescued ML-323-induced apoptosis (Fig. 3D), downregulated Noxa expression, and reduced c-PARP (cleaved-PARP) levels (Fig. 3E).

ATF4 is an important factor of ER stress; therefore, we evaluated the changes in the expression of ER stress-related proteins after ML-323 treatment. The results showed that ML-323 treatment induced the accumulation of polyubiquitinated proteins (Fig. 3F) and increased the expression of ER stress-related proteins including BIP and p-eIF2α (Fig. 3F). These results indicated that ML-323 triggers ER stress in HCC cells and induces ATF4-Noxa-mediated apoptosis.

### ML-323 induces autophagy by activating the AMPK-ULK1-ATG13 signal cascade

**Fig. 4:**
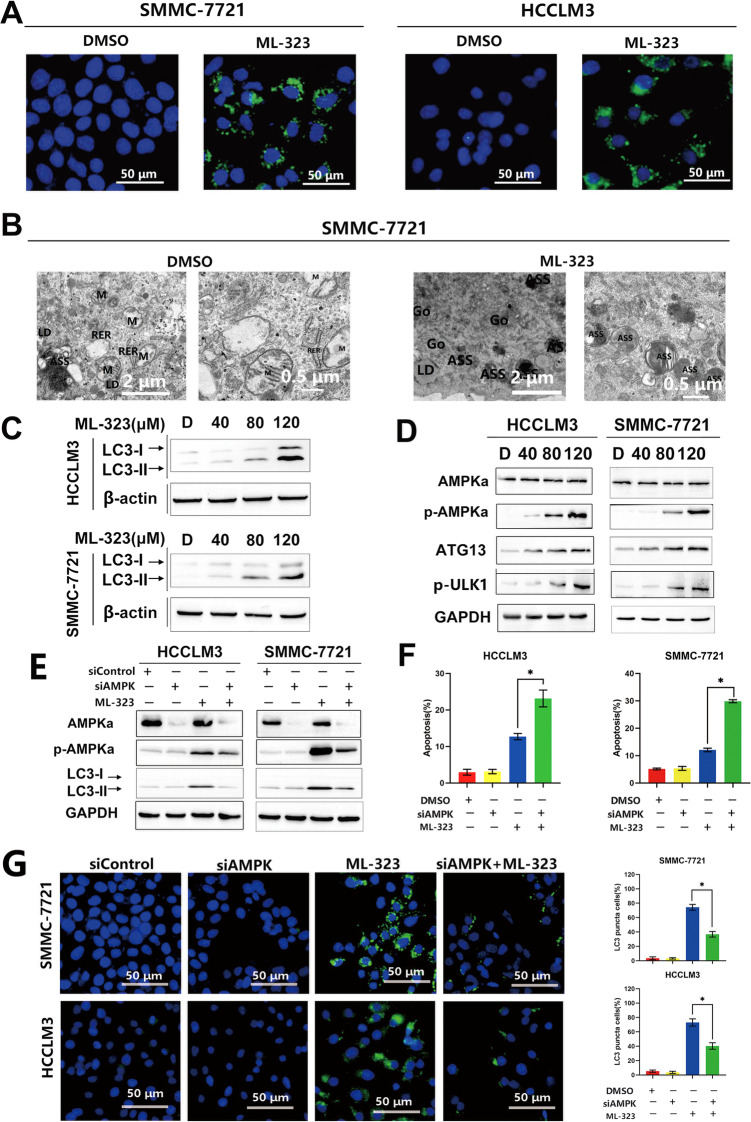
The AMPK-ATG13/ULK1 axis plays a role in ML-323-induced autophagy. **A** Immunofluorescence staining of the autophagic marker LC3B. HCCLM3 and SMMC-7721 cells were treated with ML-323 for 24 h and then incubated with LC3B primary antibody (1:200, 4 °C overnight) and Alexa Fluor 488 goat anti-rabbit IgG (H+L) secondary antibody (green) (1:500, 2 h at 25 °C), respectively. DAPI staining (blue) (5 μg/mL, 25 °C, 20 min). Images were captured using a fluorescence microscope. Representative images are shown. **B** Electron microscopic images showing the accumulation of autophagosomes in ML-323-treated HCC cells. ASS represent autophagosomes. (M: Mitochondrion, RER: Rough endoplasmic reticulum, Go: Golgi apparatus, LD: Lipid droplets). **C** Western blot analysis of LC3B expression levels in HCCLM3 and SMMC-7721 cells. **D** Western blot analysis of proteins related to the AMPK-ULK1-ATG13 signaling pathway in ML-323-treated HCCLM3 and SMMC-7721 cells. ML-323 activates the AMPK-ULK1-ATG13 signaling pathway. **E** Western blot analysis showing the effect of co-treatment with siRNA control, siAMPK, and ML-323 on protein expression. The HCCLM3 and SMMC-7721 cells were treated with ML-323 alone or in combination with siRNA control or siAMPK. **F** Annexin V-FITC /PI double-staining of AMPK-deficient HCC cells. After transfection with siControl or siAMPK, HCCLM3 and SMMC-7721 cells were treated with ML-323 for 48 h. **G** LC3B immunofluorescent staining of AMPL-deficient HCC cells shows that blocking AMPK reduced ML-323-induced autophagy. HCCLM3 and SMMC-7721 cells were treated with ML-323 alone or in combination with siRNA or siAMPK. Data represent at least three independent experiments (*n* = 3; error bars, SD).

**Fig. 5:**
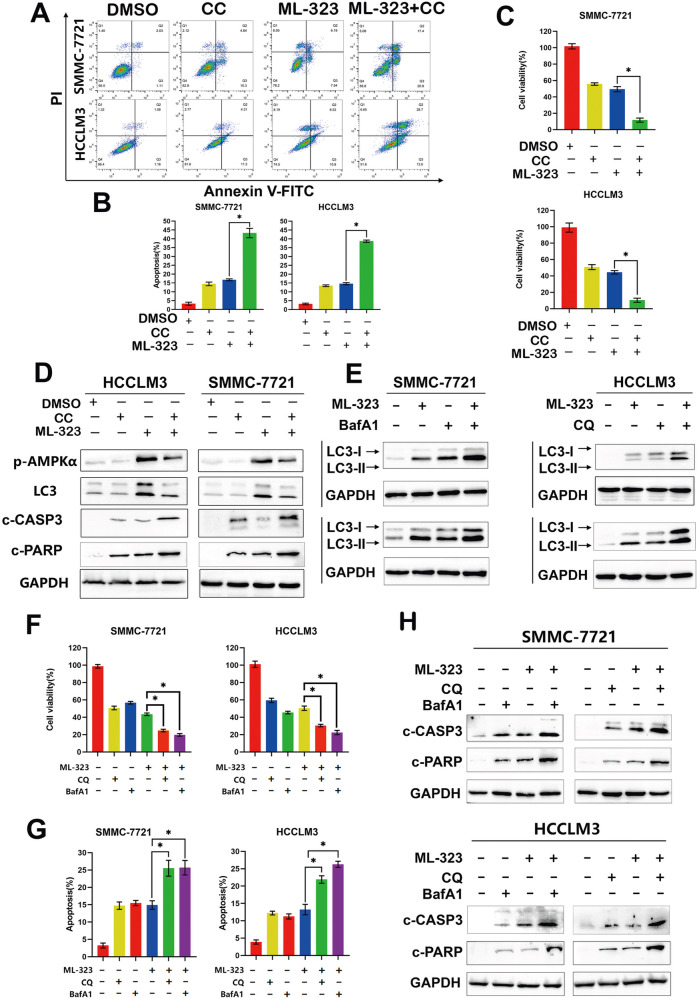
ML-323-induced increased AMPK phosphorylation triggers protective autophagy and reduces cell apoptosis. **A**, **B** Flow cytometry analysis of apoptosis was using Annexin V-FITC /PI double-staining kits. Blocking AMPK with the inhibitor CC promoted ML-323-induced apoptosis. HCCLM3 and SMMC-7721 cells were treated with ML-323 alone or in combination with DMSO or CC. Statistical analysis was conducted. **C** Cell-counting kit-8 (CCK-8) showing the cell viability of CC and ML-323-treated HCC cells. Blocking AMPK phosphorylation enhances the effect of ML-323 on HCC cell viability. HCCLM3 and SMMC-7721 cells were treated with ML-323 alone or in combination with DMSO or CC. **D** Western blot analysis of HCCLM3 and SMMC-7721 cells treated with ML-323 alone or in combination with DMSO or CC. Data represent at least three independent experiments (*n* = 3; error line, SD). **E** Western blot analysis of L3CB in HCCLM3 and SMMC-7721 cells treated with ML-323 alone, or in combination with CQ or BafA1. CQ or BafA1 inhibits LC3B degradation. **F** CCK-8 showing the cell viability of HCCLM3 and SMMC-7721 cells treated with ML-323 alone, or in combination with CQ or BafA1. Autophagy inhibition increases the ML-323 induced growth inhibition of hepatoma cells. **G** Annexin V-FITC/ PI double-staining showing the effect of the inhibition of autophagy in HCCLM3 and SMMC-7721 cells treated with ML-323 alone, or in combination with CQ or BafA1. **H** Western blot analysis of cleaved-PARP and caspase 3 in HCCLM3 and SMMC-7721 cells treated with ML-323 alone, or in combination with CQ or BafA1. Data are representative of at least three independent experiments (*n* = 3; error bars, SD).

Previous reports have indicated that DUB is involved in the regulation of autophagy31, 36; therefore, we investigated whether ML-323 can also induce autophagy in HCC cell lines by evaluating autophagic markers. As shown in Fig. 4, ML-323 treatment induced autophagy, as evidenced by positive LC3B immunofluorescence staining (Fig. 4A and Supplementary Fig. 2C), transmission electron microscopy observations of autophagosome formation (Fig. 4B), and increased LC3B expression (Fig. 4C). In addition, ML-323 promoted the phosphorylation of AMPK and ULK1 and enhanced the expression of ATG5 and ATG13 (Fig. 4D and Supplementary Fig. 5A). To further confirm the role of AMPK in ML-323-induced autophagy, HCCLM3 and SMMC-7721 cells were cotreated with ML-323 and siAMPK. After 48 h, compared with ML-323 monotherapy, co-treatment with siAMPK and ML-323 effectively downregulated the expression of p-AMPKα and attenuated ML-323-induced autophagy, as evidenced by the decreased expression of LC3B punctate-positive cells (Fig. 4G) and LC3B (Fig. 4E). Moreover, AMPK inhibition remarkably enhanced ML-323-induced cell growth inhibition (Fig. 5C) and apoptosis (Fig. 5D). These results suggest that the AMPK-ULK1-ATG13 signaling axis may play a key role in ML-323-induced autophagy.

### ML-323-induced increased AMPK phosphorylation triggers protective autophagy and reduces cell apoptosis

To further confirm the role of AMPK in ML323-induced apoptosis, HCCLM3 and SMMC-7721 cells were treated with the AMPK inhibitors CC and ML-323. After 48 h, the apoptosis rate and cell growth inhibition were detected by flow cytometry, and p-AMPKα and related apoptotic proteins (C-Casp3 and C-PARP) were detected (Fig. 5D). We found that AMPK inhibitors significantly enhanced ML-323-induced inhibition of cell growth (Fig. 5C) and promotion of apoptosis (Fig. 5A, B). After siAMPK transfected HCC cells for 48 h, cell apoptosis rate was measured, and it was found that inhibition of AMPK could increase ML-323-induced apoptosis of HCC cells. (Fig. 4F). In addition, LC3B expression was remarkably increased after co-treatment with ML-323 and the autophagy inhibitors CQ and BafA1 (Fig. 5E), indicating that CQ or BafA1 effectively blocked the later steps of ML-323-induced autophagy flux.

The inhibition of autophagy by CQ or BafA1 markedly enhanced ML-323-induced inhibition of cell viability (Fig. 5F) and promotion of apoptosis (Fig. 5G). In HCCLM3 and SMMC-7721 cells, the combination therapy with CQ or BafA1 resulted in increased levels of c-PARP and Caspase3 compared to treatment with ML-323 alone (Fig. 5H). These results suggest that ML-323 activates pro-survival autophagy and that the inhibition of autophagy significantly enhances the inhibitory effect of ML-323 on HCC cells.

### ML-323 induces ER stress in human HCC

**Fig. 6:**
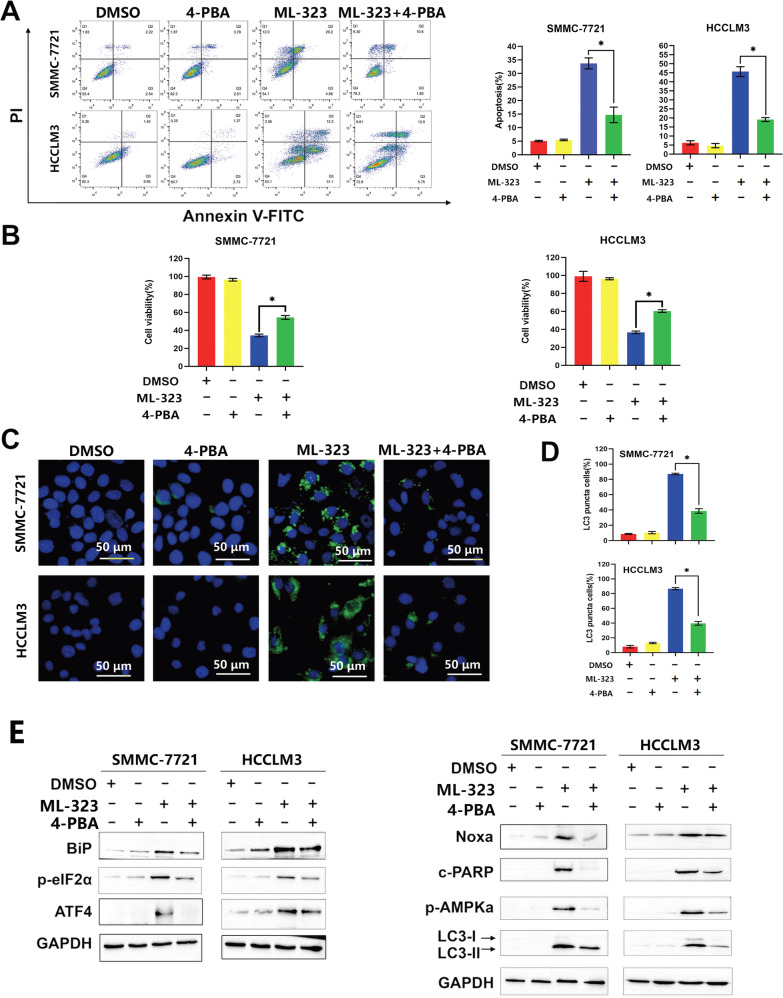
ML-323 induces ER stress in human HCC cells. **A** Flow cytometry analysis using an Annexin V-FITC/PI double-staining kit to detect apoptotic HCC cells. HCCLM3 and SMMC-7721 were cells treated with ML-323 alone or in combination with DMSO and an ER stress inhibitor (4-PBA). **B** Cell-counting kit-8 assay showing the viability of HCCLM3 and SMMC-7721 cells treated with ML-323 alone or in combination with DMSO and 4-PBA. Blocking ER stress alleviates the ML-323-induced inhibition of cell viability. **C** LC3B immunofluorescence staining in HCCLM3 and SMMC-7721 cells treated with ML-323 alone, or in combination with DMSO or 4-PBA. **D** LC3B puncta cells were statistically analyzed. **E** Western blot analysis of HCCLM3 and SMMC-7721 cells treated with ML-323 alone or in combination with DMSO or 4-PBA. Data are representative of at least three independent experiments (*n* = 3; error bars, SD). All data were representative of at least three independent experiments (*n* = 3; error bar, SD).

ML-323 treatment resulted in the accumulation of polyubiquitinated proteins and activation of ER stress (Fig. 3F). Therefore, to investigate whether ML-323 meditates its effects by inducing ER stress, HCCLM3 and SMMC-7721 cells were cotreated with ML-323 and the ER stress inhibitor 4-phenylbutyric acid (4-PBA). The results showed that 4-PBA treatment reduced cell proliferation, apoptosis (Fig. 6A, B), and autophagy (Fig. 6C, D) in ML-323-treated cells. Additionally, 4-PBA effectively inhibited ML-323-induced ER stress (Fig. 6E). According to the above results (Figs. 3E and 5D), 4-PBA downregulated the expression of ATF4-Noxa and reduced PARP cleavage and the expression of phosphorylated AMPKα and LC3B in ML-323 treated cells. Collectively, these findings suggest that ML-323 induces apoptosis and autophagy by triggering ER stress through the BIP-P-eif2α-ATF4-Noxa axis.

### Genetic inactivation of USP1 exhibits similar effects to ML-323 on HCC

**Fig. 7:**
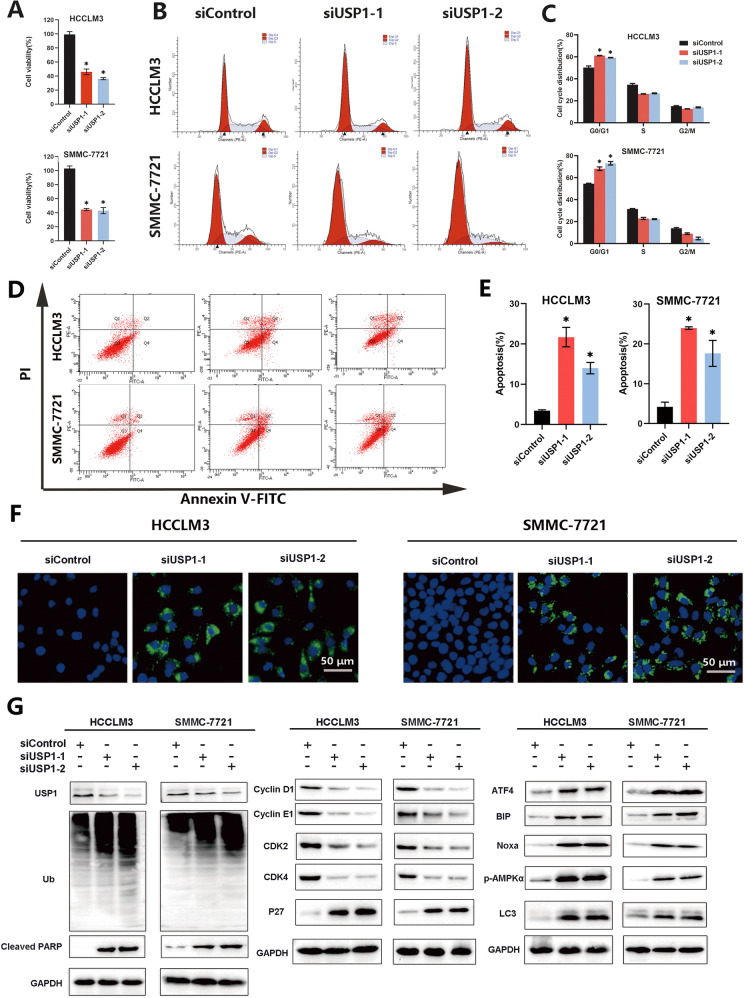
Effects of USP1 gene inactivation on HCC cells. **A** Cell-counting kit 8 assay showing the viability of HCCLM3 and SMMC-7721 cells treated with siControl and siUSP1 at 24, 48, and 72 h. **B** Flow cytometry analysis using an Annexin V-FITC/PI double-staining kit to detect apoptosis in HCCLM3 and SMMC-7721 cells treated with siControl and siUSP1 for 48 h. **C** Flow cytometry showing the cell cycle of HCCLM3 and SMMC-7721 cells treated with siControl and siUSP1 for 24 h. **D** LC3B immunofluorescence assay in HCCLM3 and SMMC-7721 cells treated with siControl and siUSP1. **E** Western blot analysis of HCCLM3 and SMMC-7721 cells treated with siControl and siUSP1. **F** GAPDH was used as a loading control. Data represent at least three independent experiments (*n* = 3; error bar, SD); scale bars = 50 μm.

To further examine the effect of USP1 inhibition on the growth of HCC cells, we used a specific siRNA to inactivate USP1 and determine whether USP1 inactivation can inhibit the growth of HCC cells. Consistent with ML-323 treatment, USP1 knockout reduced the viability of HCCLM3 and SMMC-7721 cells (Fig. 7A and Supplementary Fig. 7A). In addition, USP1 knockout induced apoptosis (Fig. 7D, E and Supplementary Fig. 7D, E), cell cycle arrest (Fig. 7B, C and Supplementary Fig. 7B, C), and autophagy (Fig. 7F and Supplementary Fig. 7F). Mechanistically, USP1 knockout also resulted in the accumulation of ubiquitinated proteins and increased the expression of cleaved-PARP, BIP, ATF4, Noxa, P27, p-AMPKα, and LC3B in HCCLM3 and SMMC-7721 cell lines (Fig. 7G and Supplementary Fig. 7G). These results suggest that the genetic inactivation of USP1 by siRNA silencing induces similar effects to those of ML-323 by promoting the apoptosis and autophagy of HCC cells.

### ML-323 inhibits tumor growth and metastasis and enhances the efficacy of sorafenib in HCC mouse models

**Fig. 8:**
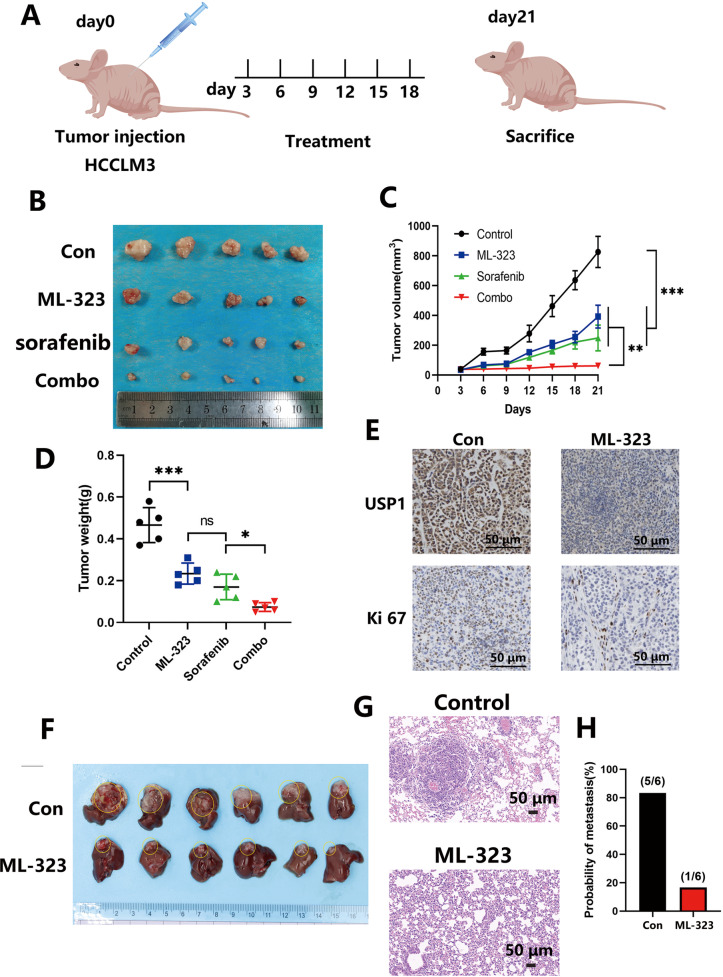
ML-323 inhibits tumor growth and enhances the efficacy of sorafenib in HCC mouse models. **A** Mice were implanted with HCC tumors and weighed starting the third day post-implantation. Tumor volume was measured in the groups once every three days. **B** ML-323 can enhance the therapeutic effect of sorafenib. HCCLM3 cells were grown into BALB/c-nu mice:(1)10%2-hydroxypropyl-β-cyclodextrin treated- mice, (2) ML-323-treated mice, (3) sorafenib-treated mice, and (4) ML-323+ sorafenib-treated mice. Tumor weights of the tumors in the four groups were recorded. **C** Tumor weights of the four groups. **D** Tumor volumes of four groups. **E** Immunohistochemical staining of tumor specimens. **F** HCCLM3 was inoculated into the orthotopic livers of nude mice and treated with ML-323 and 10%2-hydroxypropyl-β-cyclodextrin. **G** Hematoxylin and eosin (HE) staining of mouse lung tissue sections. **H** Lung metastasis of HCC in situ in mice treated with ML-323 or 10%2-hydroxypropyl-β-cyclodextrin was analyzed. Scale bars = 50 μm; *P < 0.05, **P < 0.01, ***P < 0.001, significantly different compared to the control group.

We used a human HCC subcutaneous transplantation tumor mouse model to study the anticancer potential of ML-323 in vivo and cotreated the mice with sorafenib, a commonly used chemotherapy drug for liver cancer (Fig. 8A). The results showed that ML-323 treatment effectively inhibited tumor growth and enhanced the therapeutic effect of sorafenib compared to the control group which exhibited rapid tumor growth (Fig. 8B). Tumor volume and weight were analyzed (Fig. 8C, D, *P < 0.05, **P < 0.01, ***P < 0.001). To determine the potential mechanism of ML-323, we embedded the dissected tumor to synthesize a tissue chip accordingly and verified the related proteins (USP1 and Ki 67) by IHC (Fig. 8E). In addition, HCCLM3 cells were injected into the same site on the left liver lobe of nude mice to develop orthotopic liver tumors. Four weeks after injection, the mice were euthanized and the livers were isolated from each group of mice. We found that ML-323 treatment significantly inhibited the growth of orthotopic liver tumors (Fig. 8F). Moreover, the lungs were isolated from each group of mice, and lung sections were observed by HE staining (Fig. 8G). The results indicated that ML-323 significantly inhibited the incidence of lung metastasis in the indicated orthotopic xenografts (Fig. 8H).

## Discussion

Despite advances in treating HCC, exploring potential treatment strategies is still required to improve the OS of patients with HCC. The deubiquitylating enzyme USP1 plays an important role in tumor progression; high USP1 expression has been reported in various tumors, and USP1 inhibition inhibits tumor cell growth in various cancers including lung cancer [15–25]. Therefore, USP1 inhibition is a feasible anticancer strategy. ML-323, an inhibitor of the USP1/UAF1 deubiquitinase complex [35], has been reported to have anti-tumor effects in numerous cancers including colorectal cancer [17] and breast cancer [13]. However, the mechanism by which ML-323 inhibits HCC cell growth remains largely unknown. In this study, we treated HCC cells with USP1 inhibitors, ML-323 and siUSP1, and discussed the specific mechanism of action. Our study showed that ML-323 can inhibit HCC cell proliferation by reducing cyclin D1, cyclin E1, and CDK2/4 expression, arresting the cells in the G0/G1 phase. Moreover, we revealed that ML-323 mediates its effects by inducing ER stress, thereby triggering ATF4-Noxa-mediated apoptosis by the aggregation of polyubiquitinated proteins. ML-323-induced ER stress significantly increased AMPKα phosphorylation, leading to the activation of protective autophagy. The AMPK inhibitor CC and two classic autophagy inhibitors, CQ and BafA1, further improved the therapeutic effect of ML-323 on HCC, and siAMPK had the same effect on HCC cells. Additionally, knocking down ATG5, a classic key gene that induces autophagy, also inhibited autophagy and promoted the therapeutic effects of ML-323 (Supplementary Fig. 5). Finally, we showed that ML-323 markedly inhibited HCC growth and metastasis in mice, and synergistically enhanced the therapeutic effects of sorafenib, a standard chemotherapy agent for HCC. Collectively, our results suggest that USP1 is a target and ML-323 is a potential therapeutic agent for the treatment of HCC.

USP1 is involved in a variety of cellular functions, including cell cycle regulation. Pimozide, an inhibitor of USP1, results in the arrest of adult T-cell leukemia cells in the G1 phase, leading to the accumulation of p21 and p27 and reduction in the protein levels of cyclin D2, cyclin E, CDK2, CDK4, and CDK6 [36]. This is consistent with our findings that the USP1 inhibitor ML-323 and siUSP1 treatments lead to G0/G1 arrest in HCC cells and the downregulation of the protein expression of cyclin D1, cyclin E1, CDK2, CDK4 in the initial stage of the G1 phase, in which cyclin D binds and activates CDK4 [37]. Cdk4/6 selective inhibitors result in a G1 cell cycle arrest [38]. In contrast, a previous study has reported that although shUSP1 induced showed G2/M phase arrest in colorectal cancer cells, the authors also showed that shUSP1 can down-regulate CDK4 expression [17].

In addition to changes in the expression ofCDK2 and CDK4, we also found that USP1 inhibition downregulates the expression of cyclin D1 in HCC cells. Cyclin D1 is important for G1-S transition, which requires CDK4 assembly [39]. Many therapeutic agents have been reported to downregulate cyclin D1, leading to G1 cell cycle arrest. Zhao et al. have reported that ML-323 treatment downregulated cyclin D1 and E1 protein levels [40]. Xu et al. have observed that siUSP1 reduced the expression of CCND1 and CCNE1 [17]. In addition, a recent report has shown that USP1 regulates the expression of cyclinD1 by deubiquitinating SIX1, and pharmacological or genetic inhibition of USP1 by ML-323 or siRNA downregulates the expression of SIX1 and cyclinD1, whereas the upregulation of USP1 increased the protein levels of SIX1 and cyclin D1 [41]. These results are consistent with our findings that ML-323 and siUSP1 can induce the aggregation of polyubiquitinated proteins.

ER stress is an important mechanism for DUB inhibition [31, 42]. The pro-apoptotic protein Noxa is involved in ER stress-induced cell death [43, 44]. The transcription factor ATF4 also plays an important role in Noxa activation and Noxa-mediated regulation [33]. In this study, we found that USP1 inhibition leads to the accumulation of polyubiquitinated proteins in HCC cells, triggers ER stress, and activates ATF4, and that silencing ATF4 can effectively block USP1-induced apoptosis and Noxa expression. Additionally, the ER stress inhibitor 4-PBA effectively alleviated ML-3230-induced HCC cell apoptosis by inhibiting the ER stress response and reducing Noxa expression levels, suggesting that ER stress and Noxa are at least partially involved in ML-323-induced apoptosis. Several studies have shown that ER stress can induce autophagy via the AMPK pathway [45, 46], which is consistent with our results that USP1 inhibition activated ER stress, thereby increasing the phosphorylation level of AMPK and triggering autophagy and changes in the protein levels of ATG5, ATG13, ULK1, and LC3. Our results also indicated that 4-PBA treatment can inhibit ER stress and reduce autophagy in ML-323 treated cells. Multiple lines of evidence suggest that inhibiting ER stress induces protective autophagy through AMPK-dependent pathways [47, 48]. These studies are consistent with our results that treatment with compound C blocked AMPK activation, downregulated the phosphorylation of AMPK (Thr172), and inhibited autophagy. In addition, the AMPK inhibitor, CC, effectively inhibited ML-323-induced autophagy and enhanced apoptosis. According to studies reported in authoritative literature, appropriate doses of autophagy inhibitors CQ and BafA1 were used in combination with ML-323 [49, 50]. Our results also verified that blocking autophagy with CQ or BafA1 can further enhance the inhibitory effect of USP1 on the growth of HCC cells. Therefore, our results suggest that USP1 inhibition can induce protective autophagy in HCC cells. Furthermore, the clinical drug chloroquine can inhibit autophagy in tumor cells and enhance the sensitivity of tumors to chemoradiotherapy. Previous studies have shown that combining chloroquine with traditional chemotherapy drugs can effectively improve the cure rates of breast, colon, anaplastic thyroid, and prostate cancers [50–53]. These findings are consistent with our observation that ML-323 combined with chloroquine can remarkably inhibit HCC growth, suggesting the possibility of targeting USP1 for the treatment of HCC.

In addition, USP1 inhibition can significantly inhibit the metastasis of tumor cells. For example, in breast cancer, USP1 regulates the stability of the TAZ protein through ubiquitination modification, and inhibition of USP1 can reduce the proliferation and migration of breast cancer cells [54]. USP1 interacts with KPNA2, and deubiquitination of KPNA2 is a key factor in USP1 promoting metastasis; therefore, USP1 inhibition can markedly reduce the migration of breast cancer cells [13]. A recent study has shown that USP1 can mediate the deubiquitination and stabilization of RPS16 and that ML-323 can remarkably inhibit the growth and metastasis of HCC cells [32], which is in agreement with our results. In addition, we verified the effects of ML-323 in vivo and in vitro and showed that ML-323 significantly inhibited the invasion and migration of HCC cells. Sorafenib is commonly used for the treatment of HCC with distant metastases. In our study, the combination of ML-323 with sorafenib remarkably inhibited HCC metastasis. This synergistic effect of ML-323 combined with sorafenib is possibly due to the sorafenib-induced inhibition of key cell cycle factors, including cyclin D1, cyclin E1, and CDKs [55–57]. Sorafenib sensitivity to HCC is related to the expression of cyclin E1; inhibition of cyclin E1 promoted sorafenib-induced apoptosis of HCC cells [58]. Moreover, the addition of the generalized CDK inhibitor flavopiridol greatly improved the efficacy of sorafenib in vivo and in vitro [58]. Therefore, we believe that targeting ML-323 or USP1 can increase the sensitivity of HCC cells to sorafenib treatment by inhibiting the expression of cyclin E1, cyclin D1, CDK2, and CDK4.

**Fig. 9:**
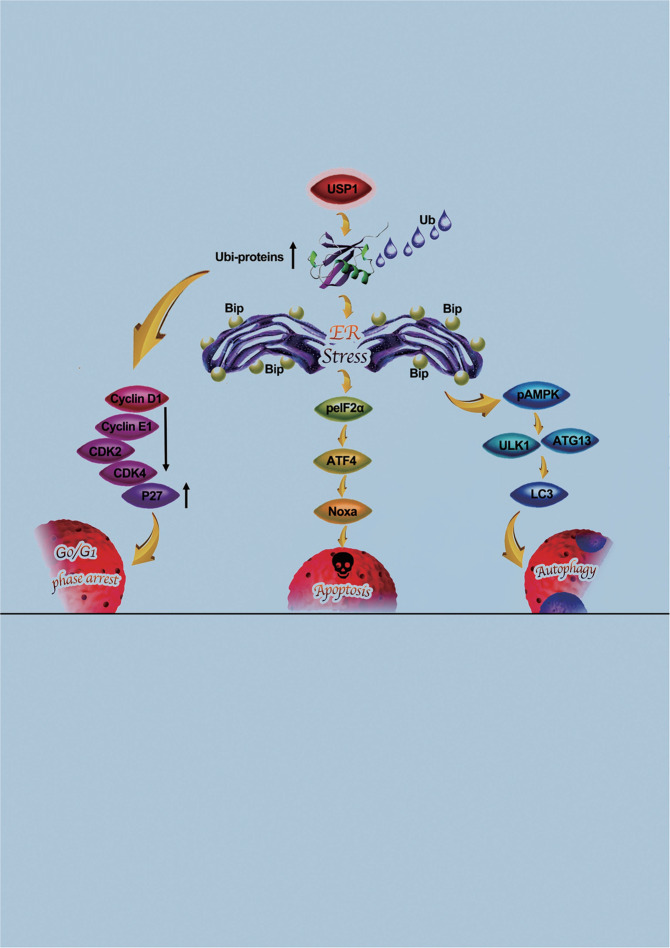
This paper studies signal machine drawing. Schema of the mechanism for targeting USP1 induced cell cycle arrest, apoptosis and autophagy in HCC.

Equally important, we found that USP1 inhibitor ML-323 combined with autophagy inhibitor CQ and clinical chemotherapy drug sorafenib could better inhibit the growth of HCC (Supplementary Fig. 8). Therefore, targeting USP1 in combination with autophagy inhibitors and sorafenib may be an attractive therapeutic strategy for HCC. Collectively, this study revealed the detailed mechanism of targeting USP1 for HCC cells (Fig. 9). Our findings provide a strong impetus for the clinical investigation of targeting USP1 for the treatment of HCC.

In summary, this study provides a new understanding of the inhibitory effect and cytotoxic mechanism of USP1 inhibitors on HCC cell proliferation, suggesting that targeting USP1 may be a potential therapeutic strategy for HCC.

## Supplementary information

Supplementary Figure 1

Supplementary Figure 2

Supplementary Figure 3

Supplementary Figure 4

Supplementary Figure 5

Supplementary Figure 6

Supplementary Figure 7

Supplementary Figure 8

SUPPLEMENTAL MATERIAL

Original image(WB)

Reproducibility checklist

## Data Availability

All data generated or analyzed during this study are available from the corresponding author upon reasonable request.
