# Supplementary figures and images for: Inhibition of USP1 activates ER stress through Ubi-protein aggregation to induce autophagy and apoptosis in HCC
Source: Cell Death Dis. 2022 Nov 10;13(11):951. doi: 10.1038/s41419-022-05341-3 (PMC9649627; doi:10.1038/s41419-022-05341-3)

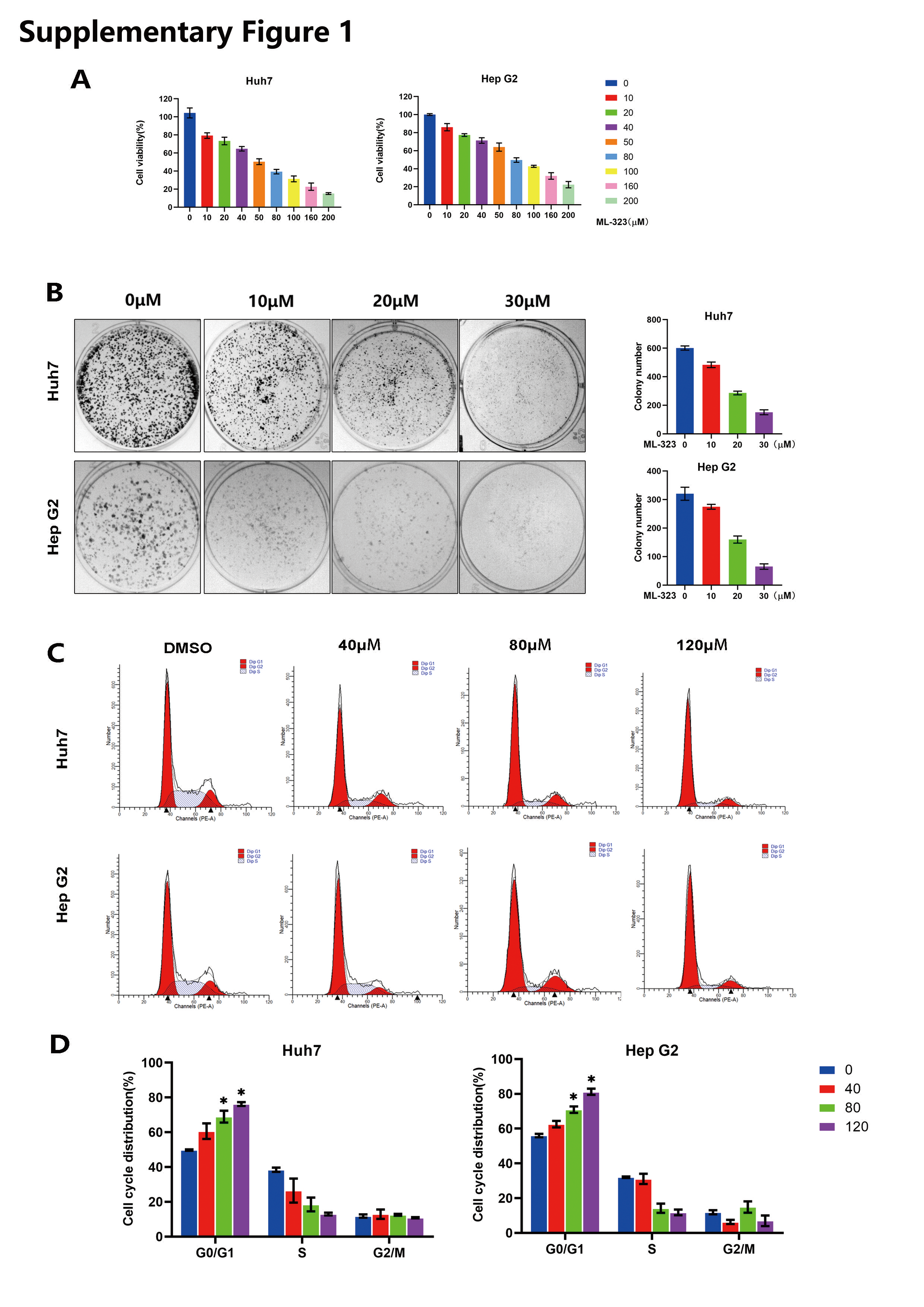

Supplement: Supplementary file 1 — Supplementary Figure 1 [file 41419_2022_5341_MOESM1_ESM.png]

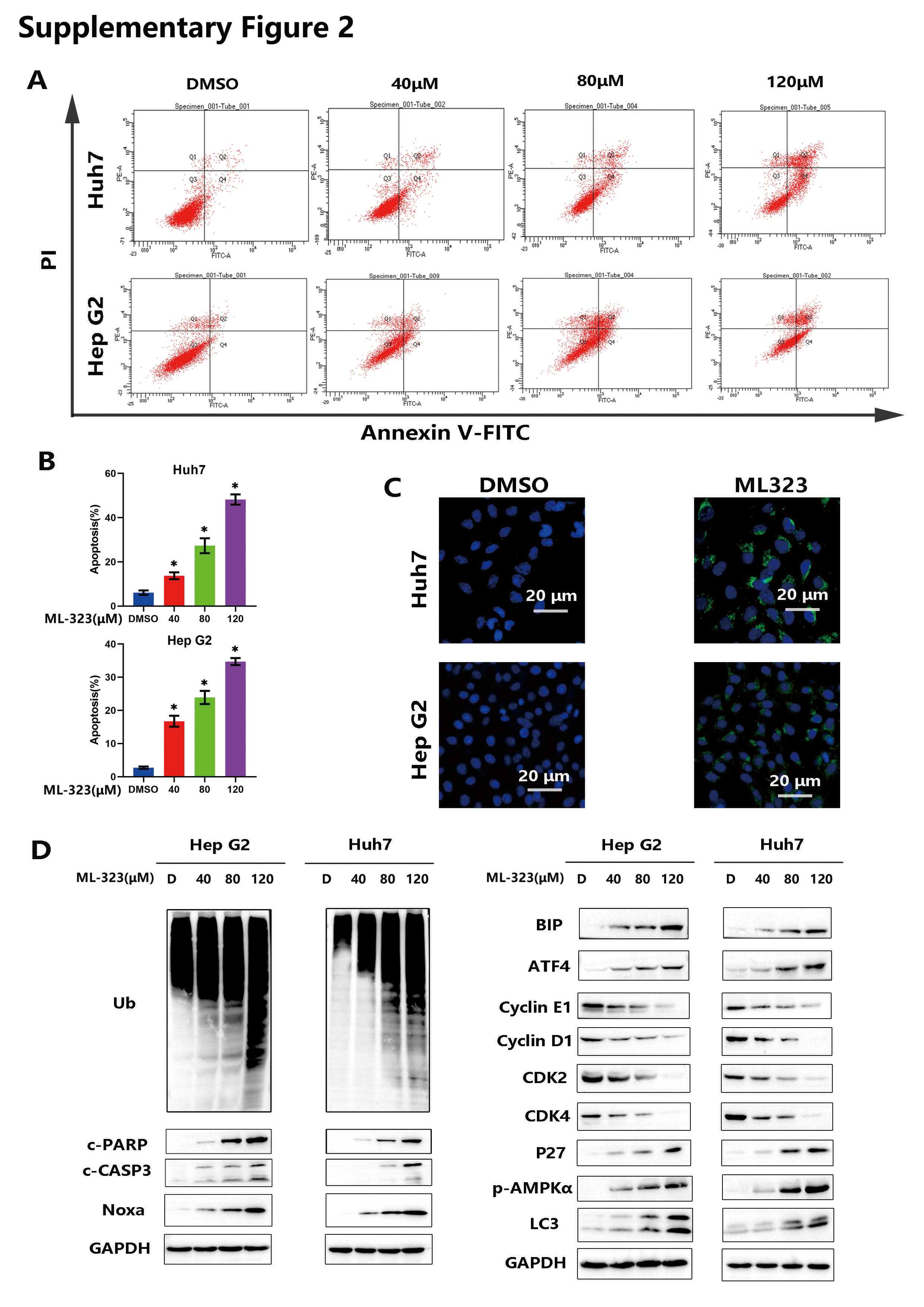

Supplement: Supplementary file 2 — Supplementary Figure 2 [file 41419_2022_5341_MOESM2_ESM.png]

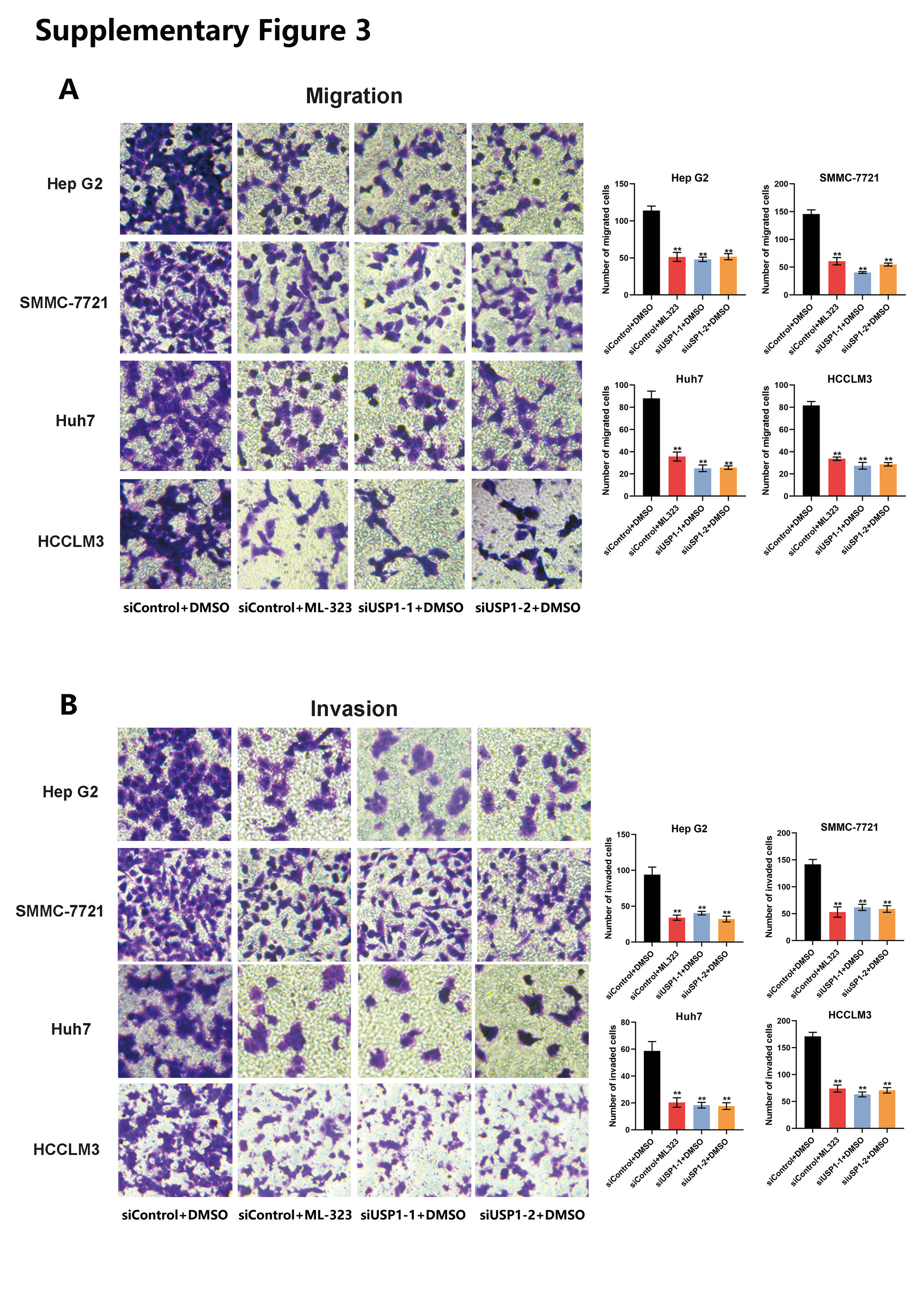

Supplement: Supplementary file 3 — Supplementary Figure 3 [file 41419_2022_5341_MOESM3_ESM.png]

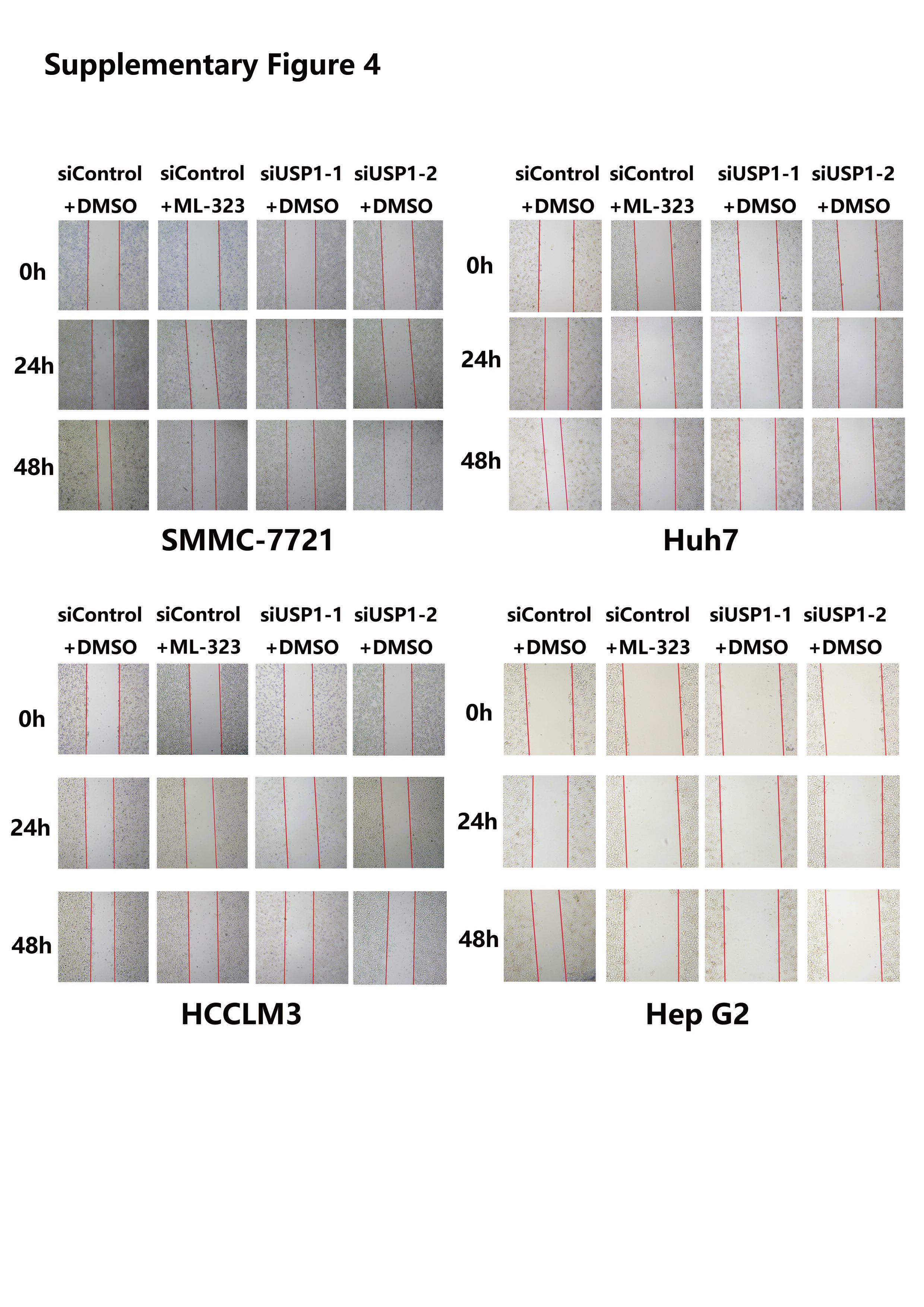

Supplement: Supplementary file 4 — Supplementary Figure 4 [file 41419_2022_5341_MOESM4_ESM.png]

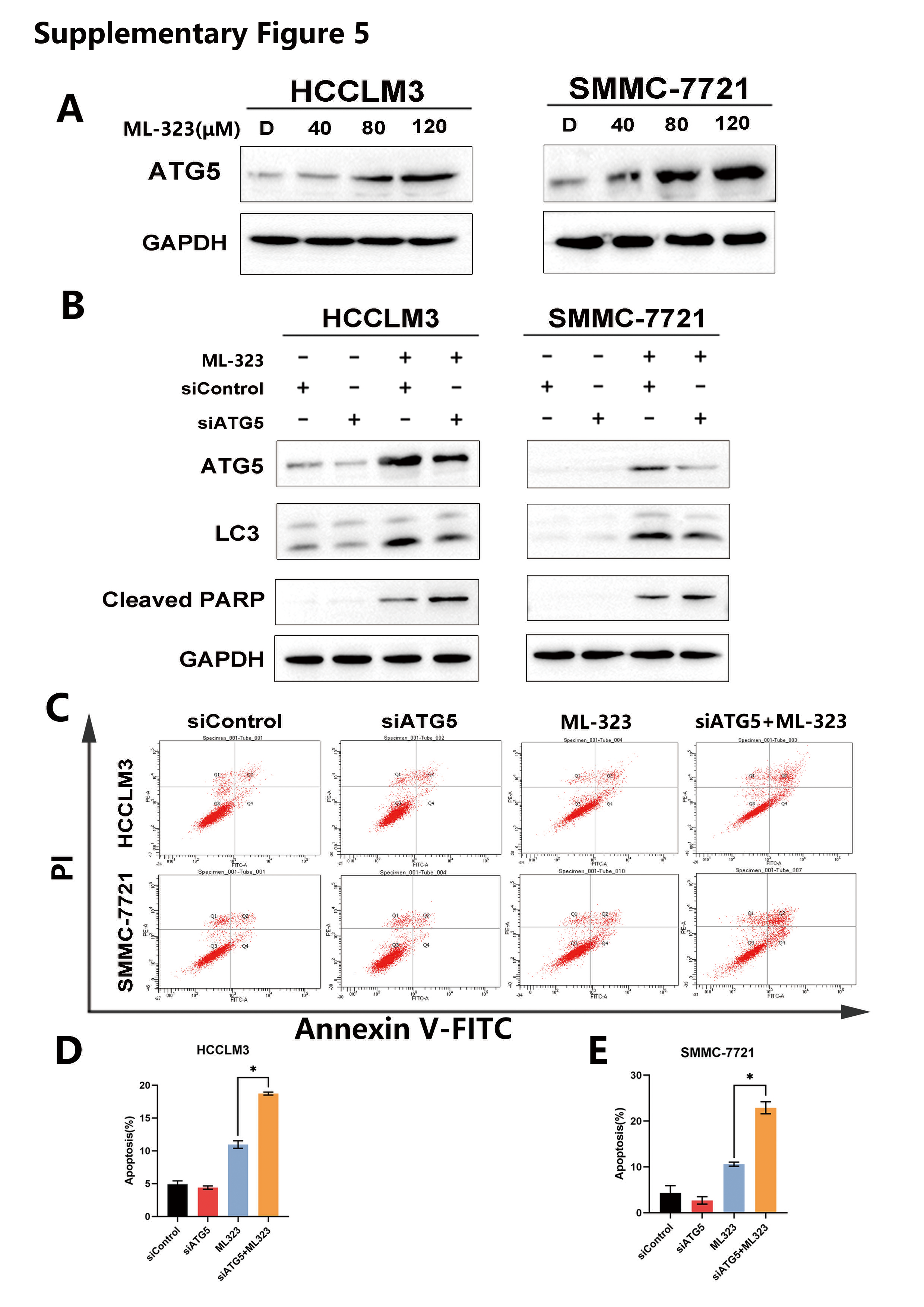

Supplement: Supplementary file 5 — Supplementary Figure 5 [file 41419_2022_5341_MOESM5_ESM.png]

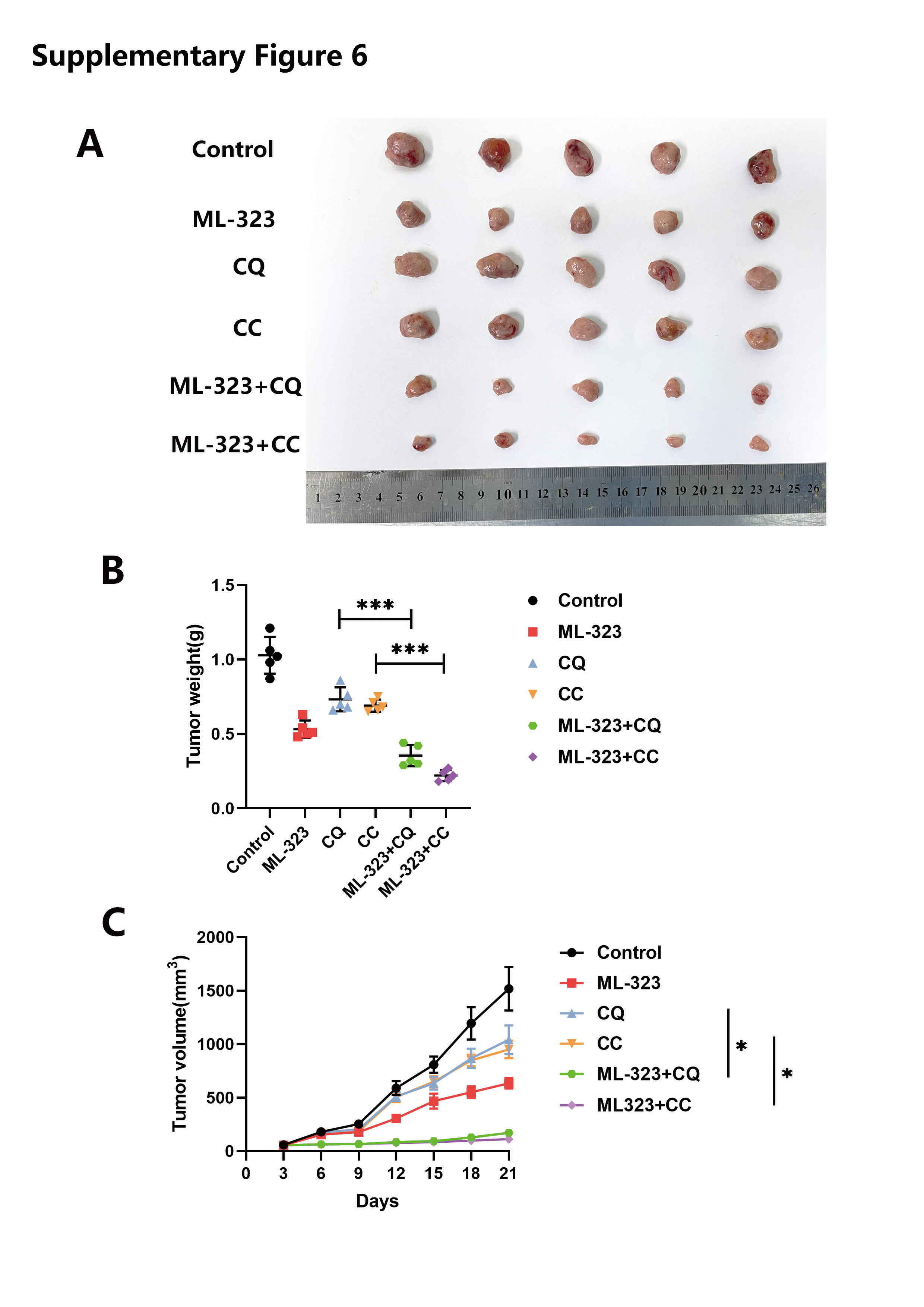

Supplement: Supplementary file 6 — Supplementary Figure 6 [file 41419_2022_5341_MOESM6_ESM.png]

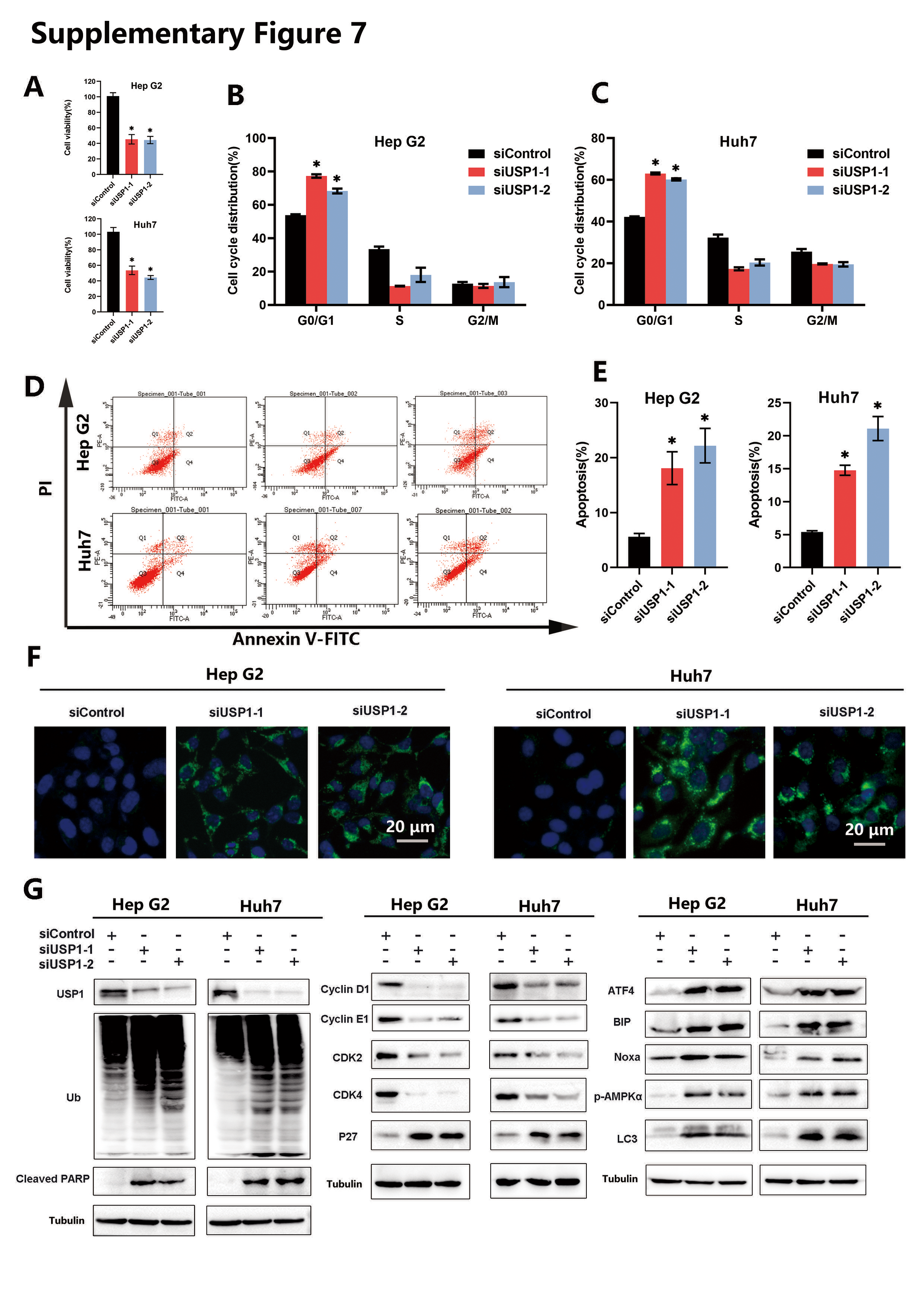

Supplement: Supplementary file 7 — Supplementary Figure 7 [file 41419_2022_5341_MOESM7_ESM.png]

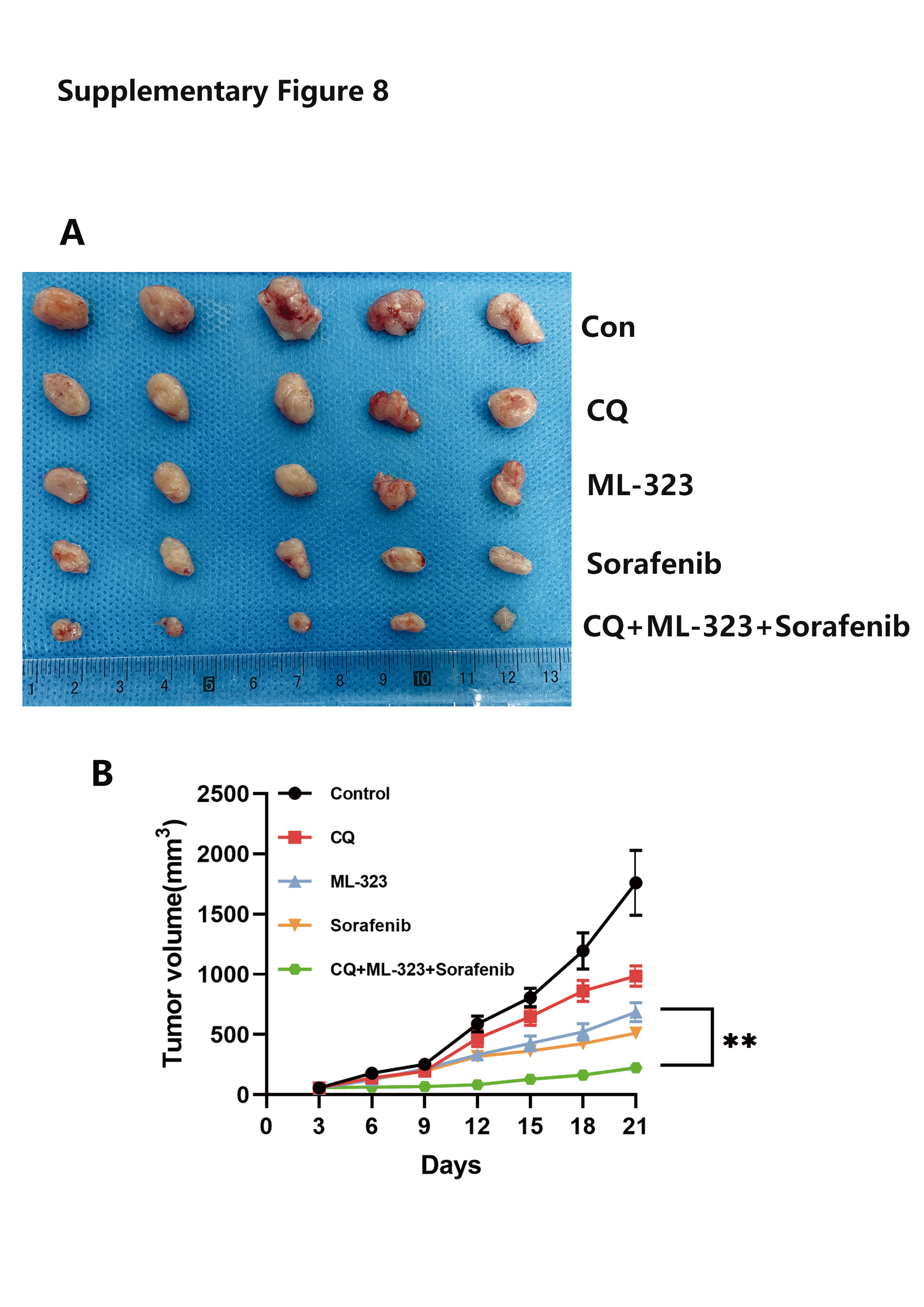

Supplement: Supplementary file 8 — Supplementary Figure 8 [file 41419_2022_5341_MOESM8_ESM.png]
