# Supplementary material for: Inhibition of USP1 activates ER stress through Ubi-protein aggregation to induce autophagy and apoptosis in HCC
Source: Cell Death Dis. 2022 Nov 10;13(11):951. doi: 10.1038/s41419-022-05341-3 (PMC9649627; doi:10.1038/s41419-022-05341-3)
Supplement: Supplementary file 9 — SUPPLEMENTAL MATERIAL [file 41419_2022_5341_MOESM9_ESM.docx]

Supplementary Table S1

Table S1: Detailed information of tissue array used in IHC

| NO. | Gender | Age | TNM | Histologic grade | NO. | Gender | Age | TNM | Histologic grade |
| --- | --- | --- | --- | --- | --- | --- | --- | --- | --- |
| 1 | M | 49 | Ⅲ | 1 | 41 | F | 53 | Ⅰ | 2 |
| 2 | M | 43 | Ⅱ | 1 | 42 | F | 61 | Ⅰ | 1 |
| 3 | M | 49 | Ⅱ | 1 | 43 | M | 58 | Ⅰ | 1-2 |
| 4 | M | 46 | Ⅰ | 1-2 | 44 | M | 48 | Ⅰ | 1-2 |
| 5 | M | 49 | Ⅰ | 2 | 45 | M | 45 | Ⅱ | 1 |
| 6 | M | 66 | Ⅲ | 3 | 46 | M | 62 | Ⅱ | 2 |
| 7 | M | 42 | Ⅰ | 1-2 | 47 | M | 50 | Ⅰ | 1 |
| 8 | M | 66 | Ⅰ | 1 | 48 | M | 58 | Ⅰ | 1-2 |
| 9 | M | 37 | Ⅰ | 3 | 49 | M | 57 | Ⅰ | 1 |
| 10 | M | 58 | Ⅱ | 1-2 | 50 | M | 43 | Ⅱ | 1-2 |
| 11 | M | 62 | Ⅱ | 1-2 | 51 | M | 66 | Ⅰ | 1-2 |
| 12 | M | 32 | Ⅰ | 1 | 52 | F | 61 | Ⅰ | 1 |
| 13 | M | 54 | Ⅰ | 2 | 53 | M | 46 | Ⅰ | 2 |
| 14 | M | 75 | Ⅰ | 1-2 | 54 | M | 68 | Ⅱ | 1-2 |
| 15 | M | 47 | Ⅰ | 3 | 55 | M | 60 | Ⅲ | 2 |
| 16 | M | 67 | Ⅰ | 1 | 56 | M | 53 | Ⅰ | 1-2 |
| 17 | F | 62 | Ⅲ | 1 | 57 | M | 57 | Ⅰ | 3 |
| 18 | M | 64 | Ⅰ | 2 | 58 | M | 65 | Ⅲ | 1 |
| 19 | F | 46 | Ⅱ | 1-2 | 59 | M | 49 | Ⅲ | 1-2 |
| 20 | M | 56 | Ⅲ | 1 | 60 | M | 60 | Ⅰ | 1-2 |
| 21 | M | 50 | Ⅰ | 1-2 | 61 | M | 71 | Ⅰ | 2 |
| 22 | M | 61 | Ⅱ | 1 | 62 | F | 66 | Ⅰ | 1 |
| 23 | M | 66 | Ⅲ | 2-3 | 63 | F | 52 | Ⅰ | 1-2 |
| 24 | M | 49 | Ⅰ | 1 | 64 | M | 49 | Ⅰ | 3 |
| 25 | F | 60 | Ⅰ | 1-2 | 65 | F | 68 | Ⅰ | 2-3 |
| 26 | F | 37 | Ⅰ | 2 | 66 | F | 45 | Ⅱ | 2-3 |
| 27 | M | 50 | Ⅰ | 1-2 | 67 | M | 47 | Ⅰ | 1 |
| 28 | M | 47 | Ⅰ | 1 | 68 | M | 52 | Ⅰ | 1-2 |
| 29 | F | 60 | Ⅰ | 2-3 | 69 | M | 43 | Ⅰ | 1-2 |
| 30 | M | 55 | Ⅲ | 2 | 70 | F | 43 | Ⅲ | 1 |
| 31 | M | 46 | Ⅰ | 2 | 71 | M | 67 | Ⅰ | 1-2 |
| 32 | M | 53 | Ⅰ | 2 | 72 | M | 62 | Ⅰ | 1 |
| 33 | F | 63 | Ⅰ | 1 | 73 | F | 48 | Ⅱ | 1-2 |
| 34 | F | 60 | Ⅰ | 1 | 74 | M | 63 | Ⅰ | 1-2 |
| 35 | M | 72 | Ⅰ | 1-2 | 75 | F | 54 | Ⅱ | 1-2 |
| 36 | M | 57 | Ⅰ | 1-2 | 76 | M | 50 | Ⅰ | 1-2 |
| 37 | M | 55 | Ⅰ | 1-2 | 77 | M | 71 | Ⅰ | 1-2 |
| 38 | M | 51 | Ⅰ | 1-2 | 78 | M | 52 | Ⅲ | 2-3 |
| 39 | M | 74 | Ⅲ | 1-2 | 79 | M | 56 | Ⅰ | 2-3 |
| 40 | M | 76 | Ⅰ | 2 | 80 | M | 59 | Ⅰ | 3 |
