# Supplementary material for: Inhibition of USP1 activates ER stress through Ubi-protein aggregation to induce autophagy and apoptosis in HCC
Source: Cell Death Dis. 2022 Nov 10;13(11):951. doi: 10.1038/s41419-022-05341-3 (PMC9649627; doi:10.1038/s41419-022-05341-3)
Supplement: Supplementary file 10 — Original image(WB) [file 41419_2022_5341_MOESM10_ESM.pdf]

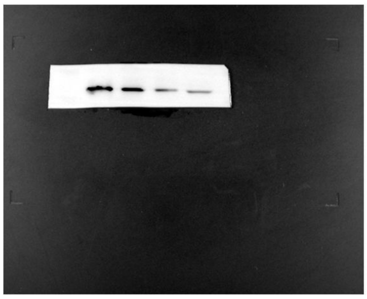
CDK2

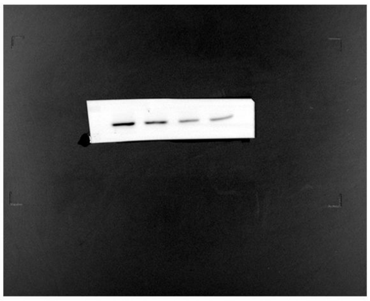
CDK2

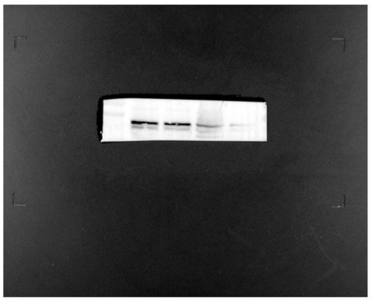
CYCLIN E1

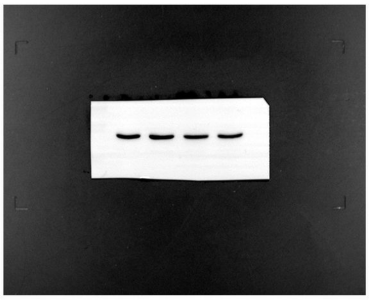
GAPDH

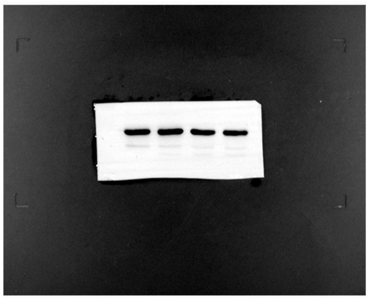
GAPDH

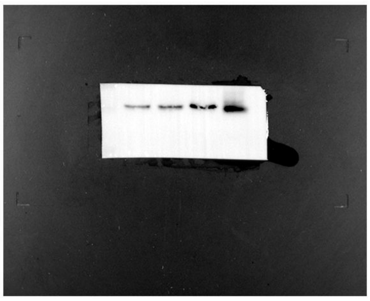
P27

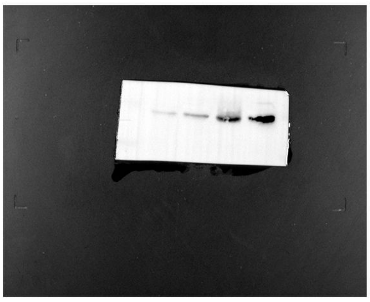
P27

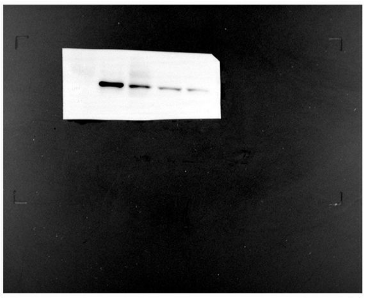
CDK4

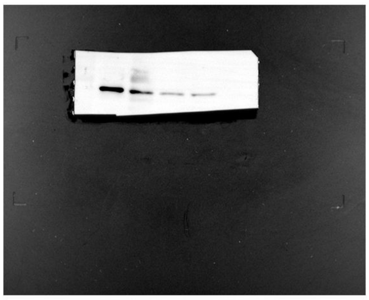
CDK4

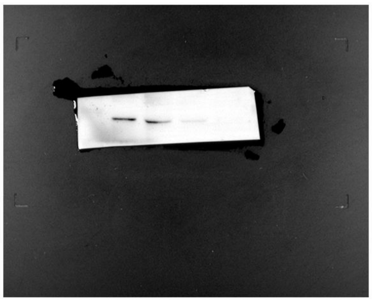
CYCLIN D1

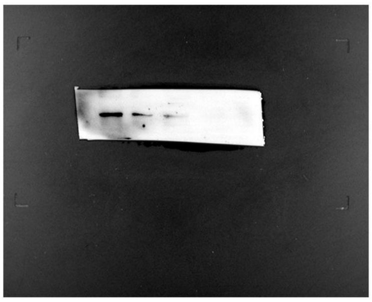
CYCLIN D1

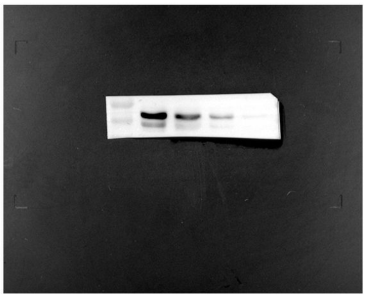
CYCLIN E1

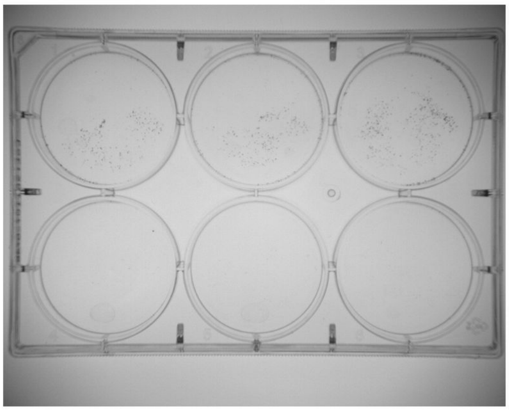

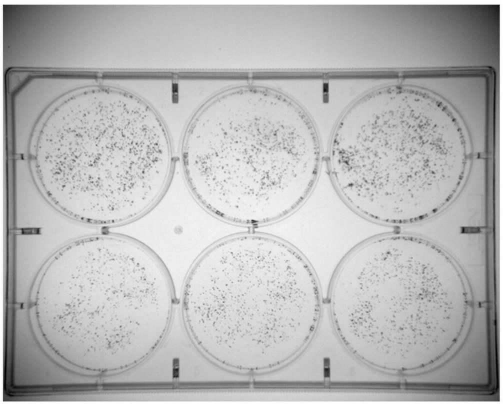

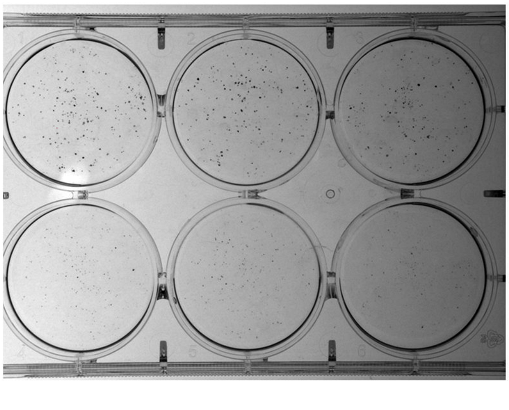

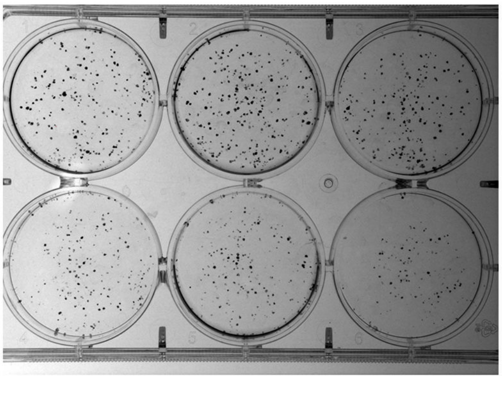

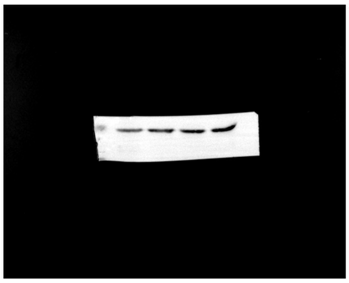

BAK

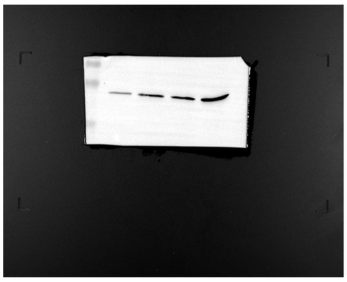

BAK

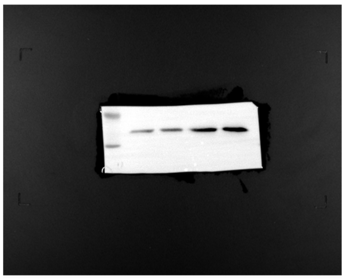

BAX

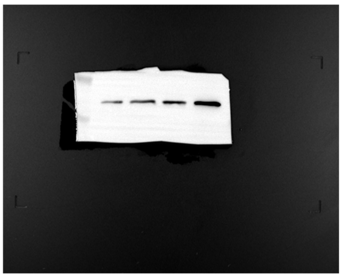

BAX

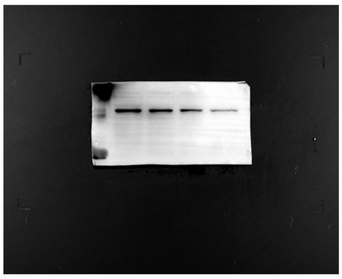

BCL-2

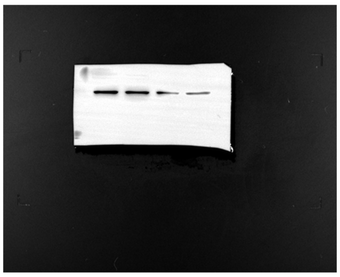

BCL-2

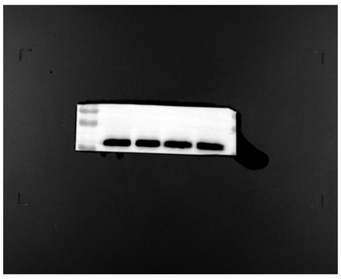

BCL-XL

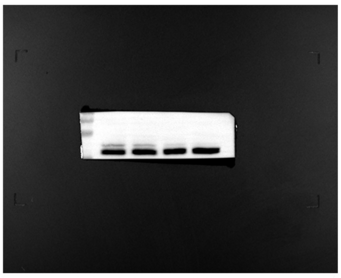

BCL-XL

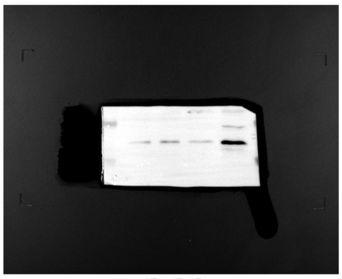

CC3

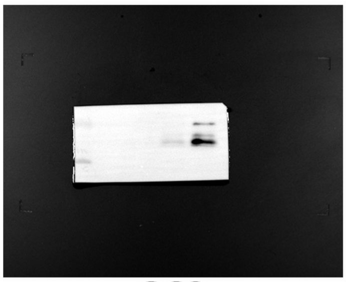

CC3

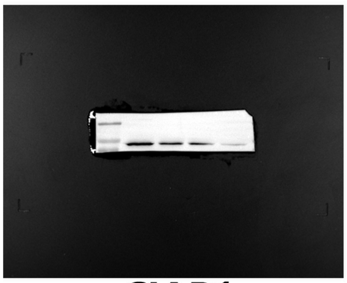

CIAP1

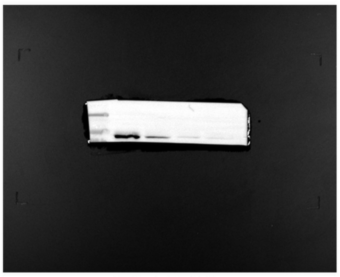

CIAP1

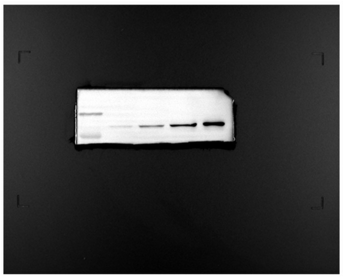

CP

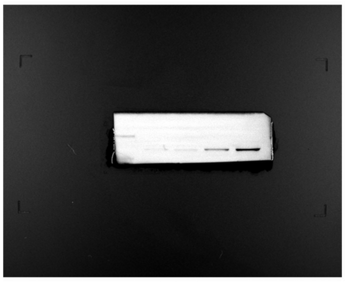

CP

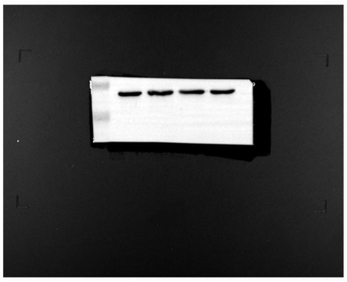

GAPDH

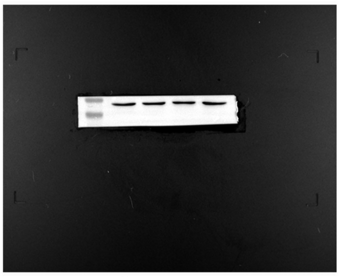

GAPDH

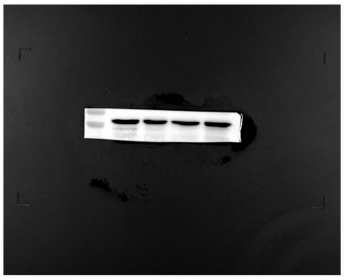

MCL-1

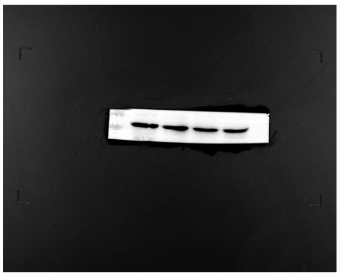

MCL-1

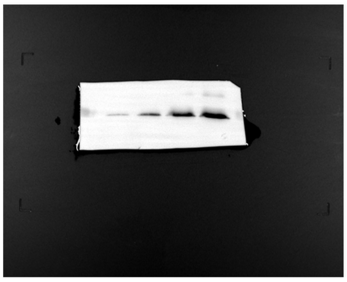

NOXA

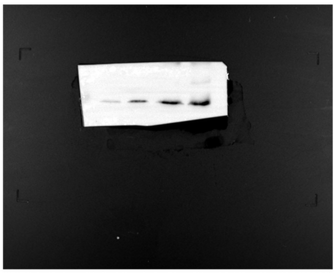

NOXA

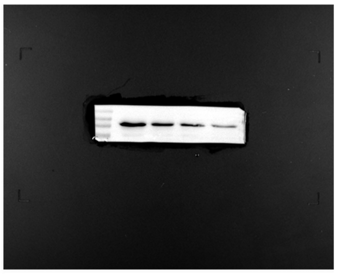

XIAP

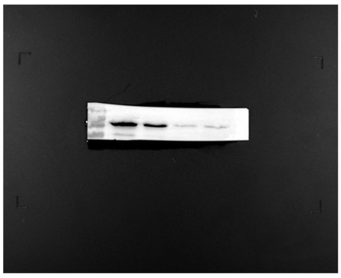

XIAP

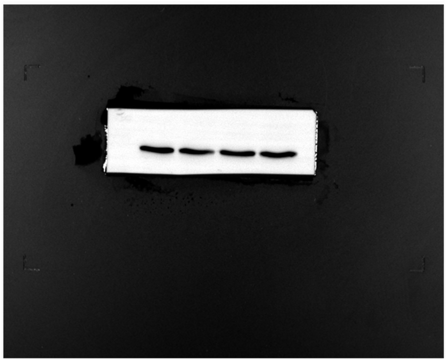

**β-actin**

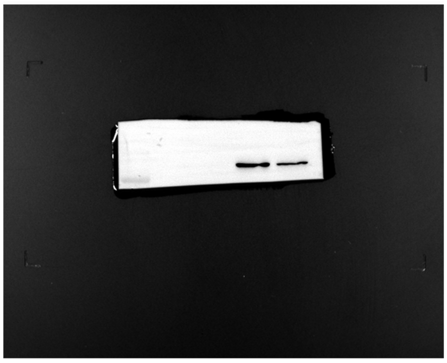

**CP**

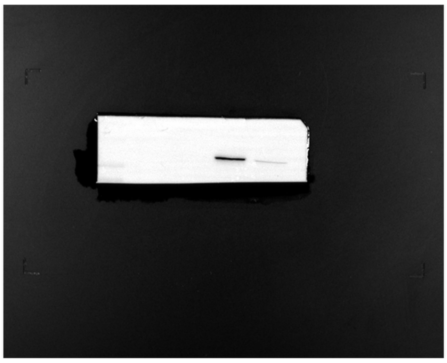

**CP**

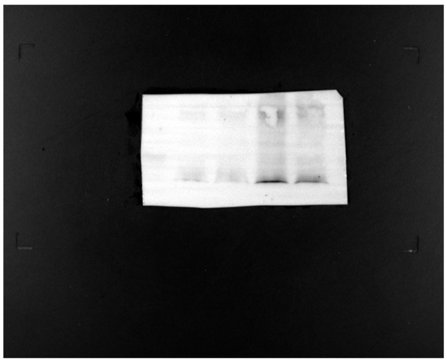

**NOXA**

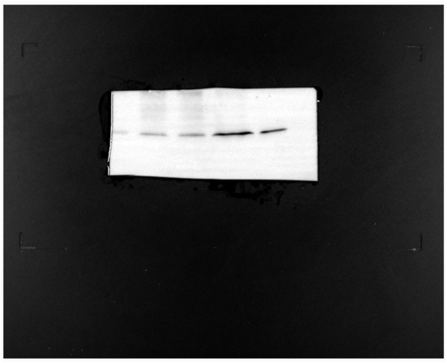

**NOXA**

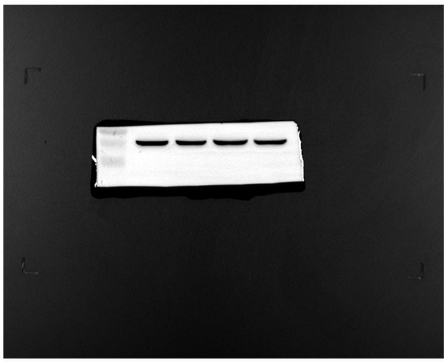

**β-actin**

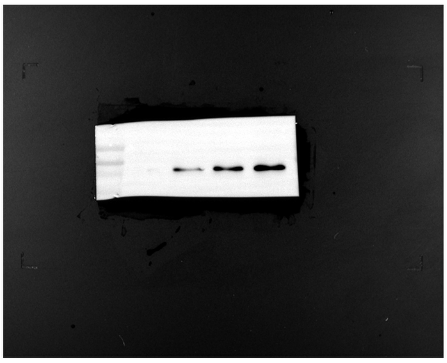

ATF4

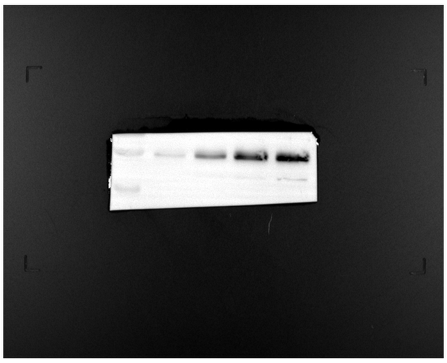

ATF4

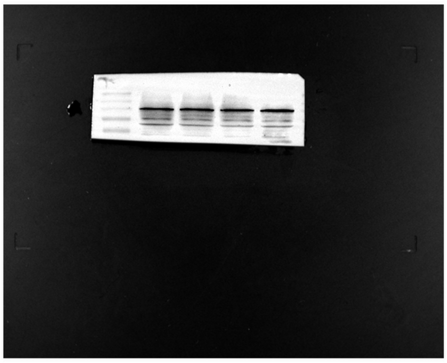

Hif-1α

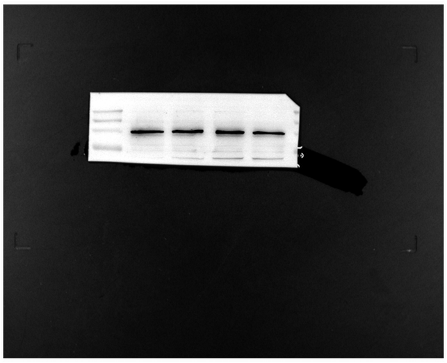

Hif-1α

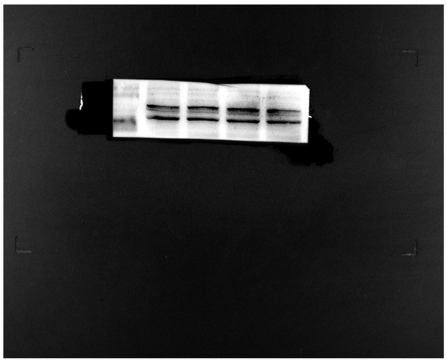

Foxo3a

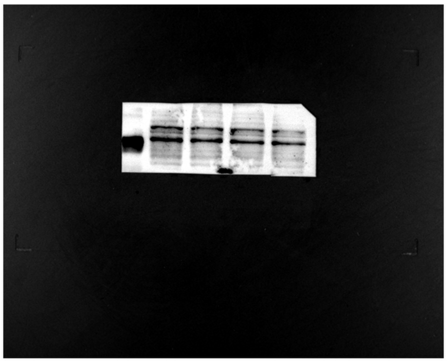

Foxo3a

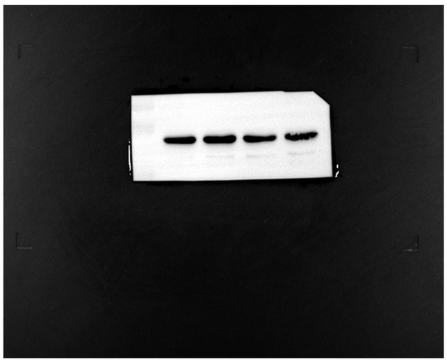

GAPDH

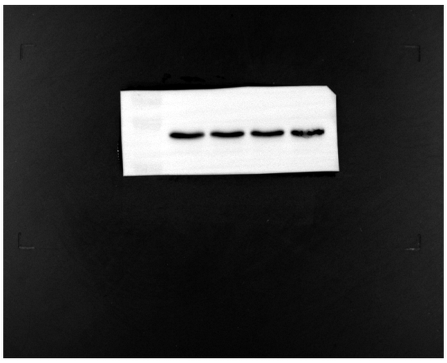

GAPDH

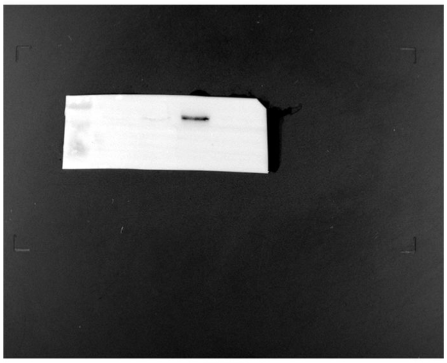
ATF4

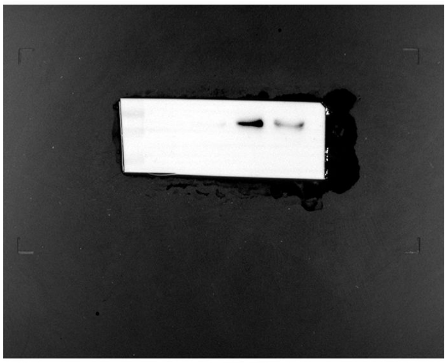
ATF4

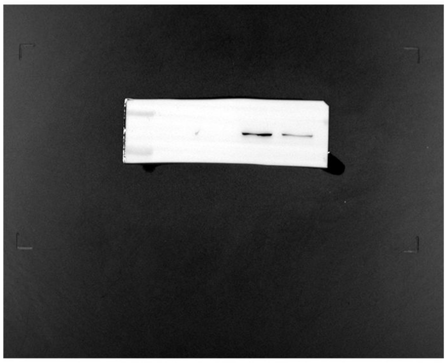
CLEAVED-PARP

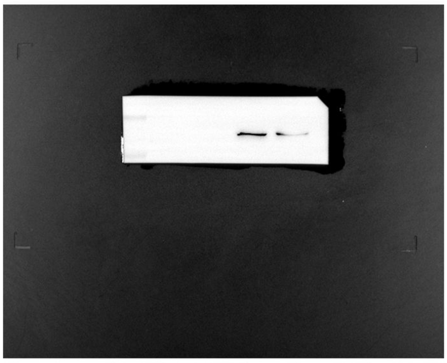
CLEAVED-PARP

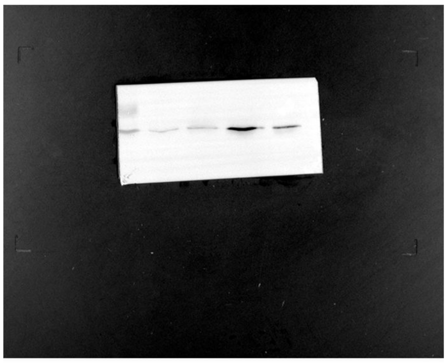
Noxa

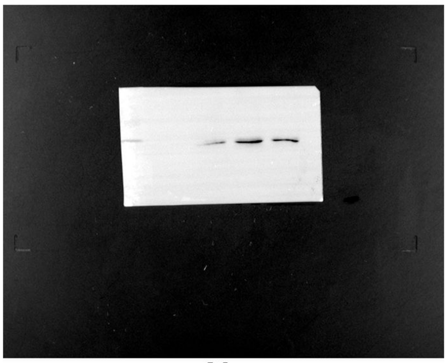
Noxa

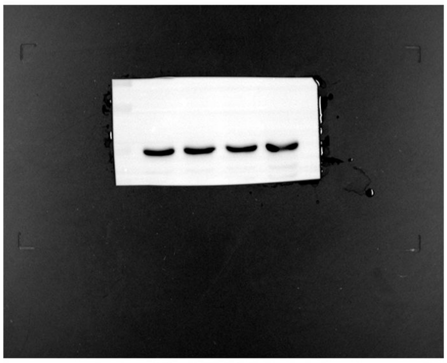
GAPDH

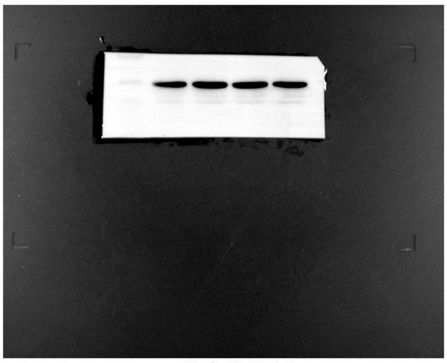
GAPDH

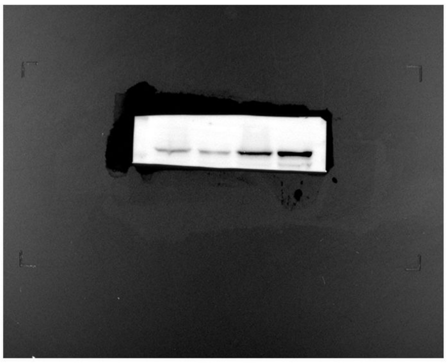

BIP

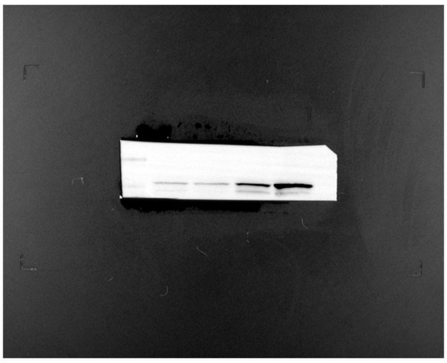

BIP

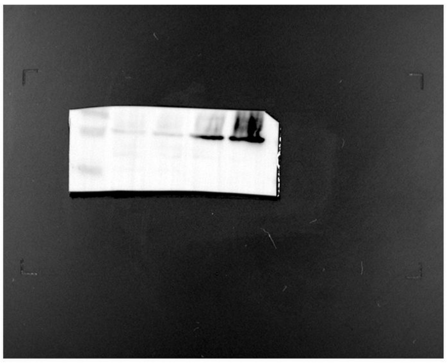

P-eif2α

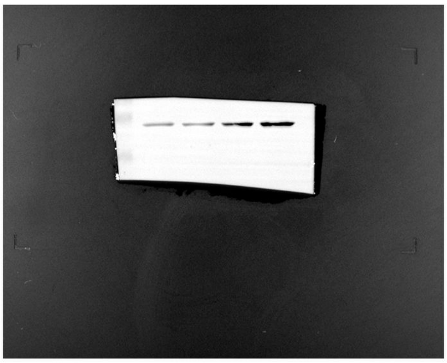

P-eif2α

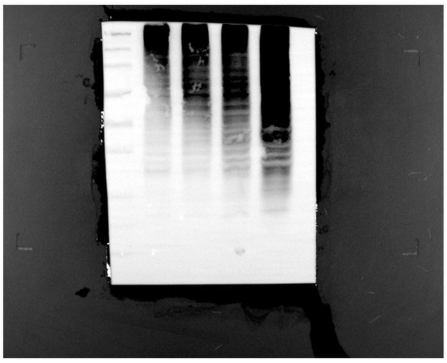

Ub

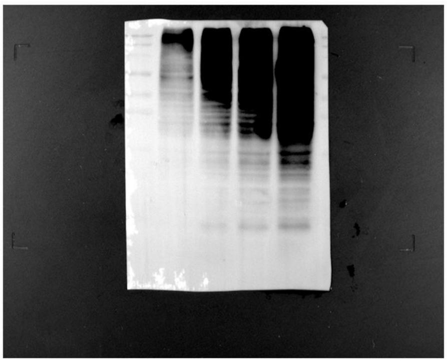

Ub

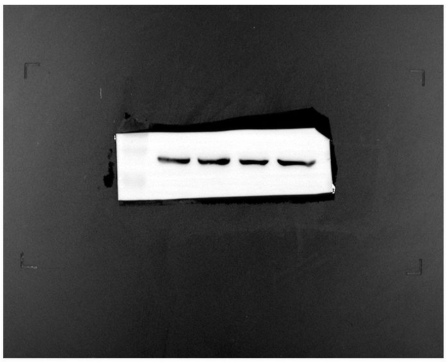

β-actin

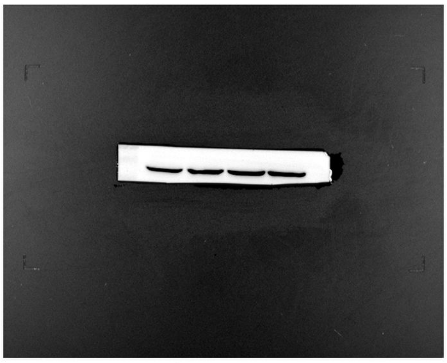

β-actin

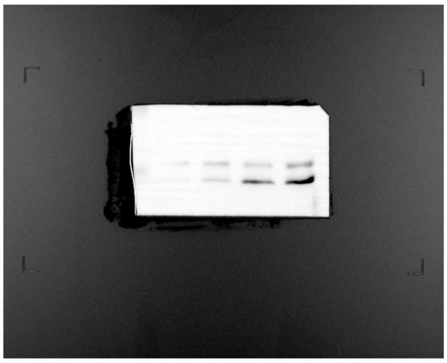

LC3

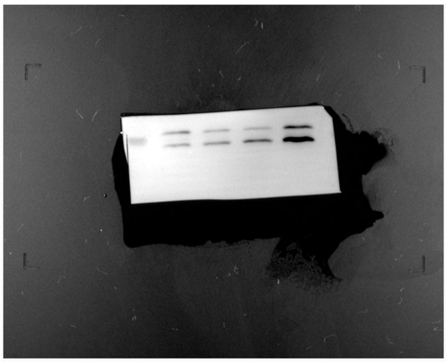

LC3

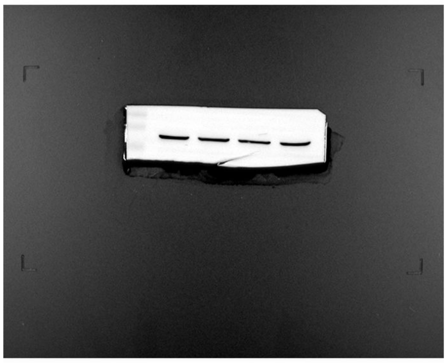

β-actin

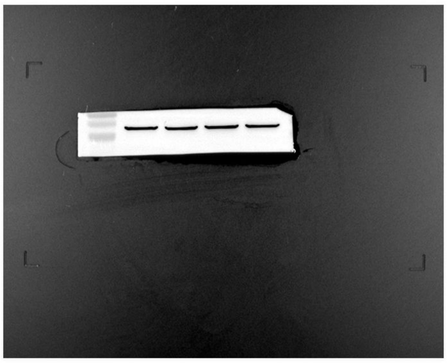

β-actin

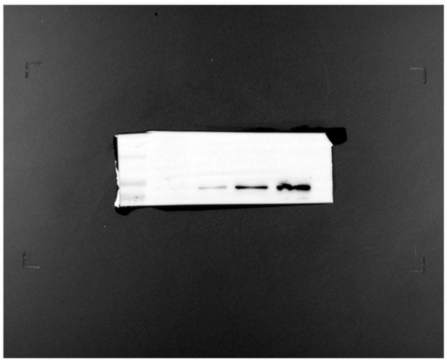
P-AMPK α

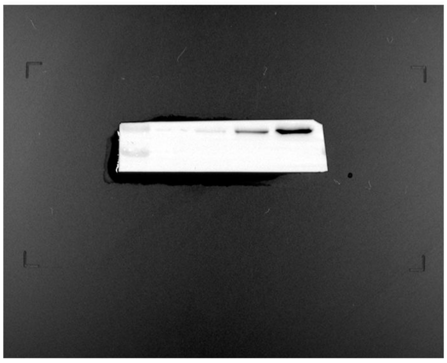
P-AMPK α

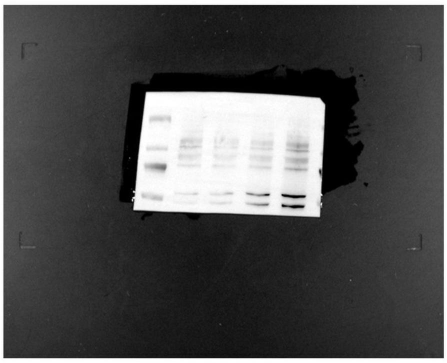
P-ULK1

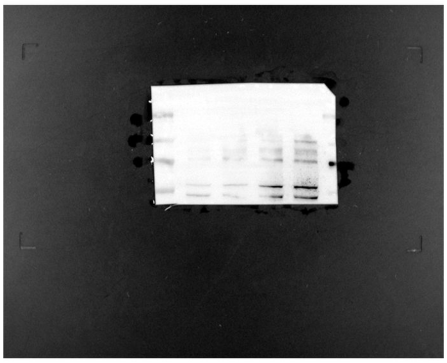
P-ULK1

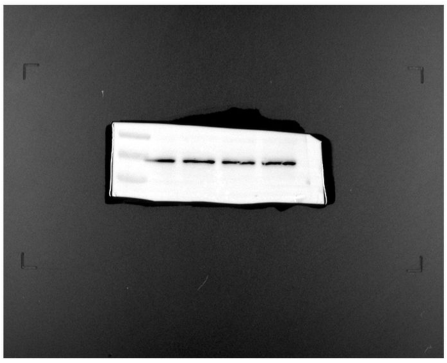
AMPK α

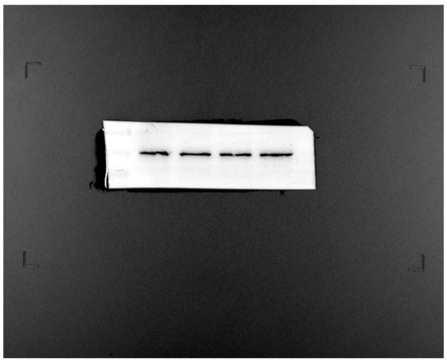
AMPK α

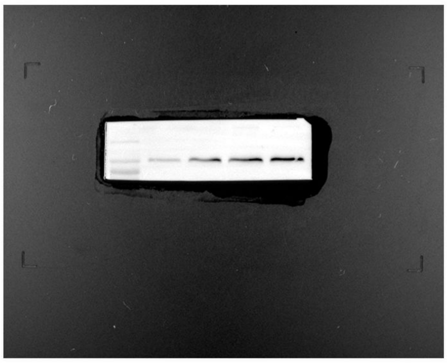
ATG13

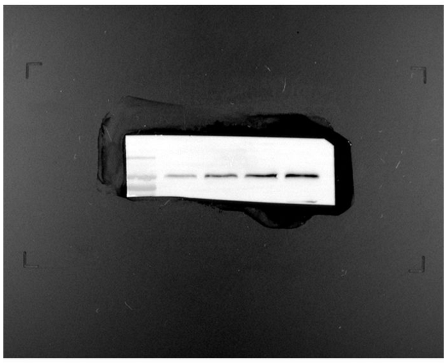
ATG13

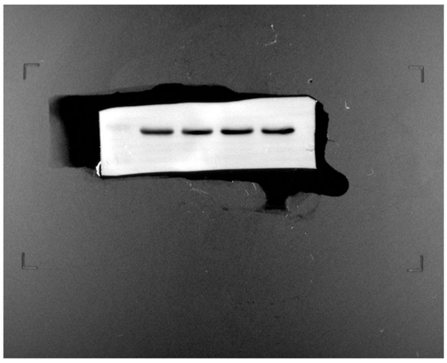
GAPDH

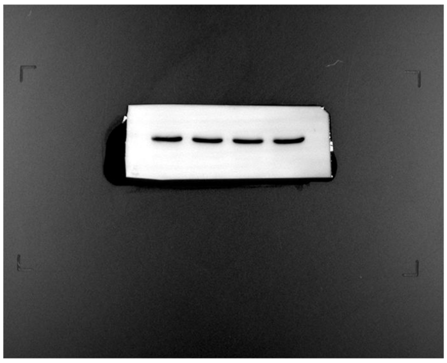
GAPDH

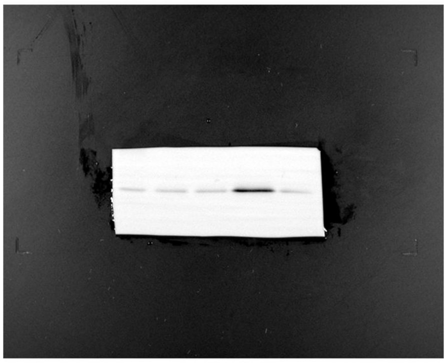

LC3

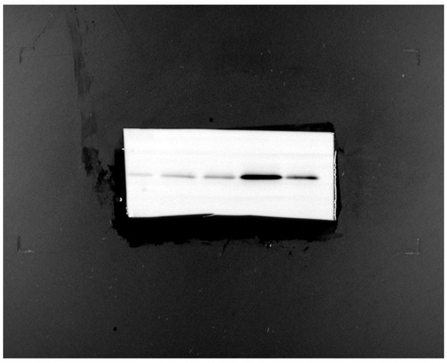

LC3

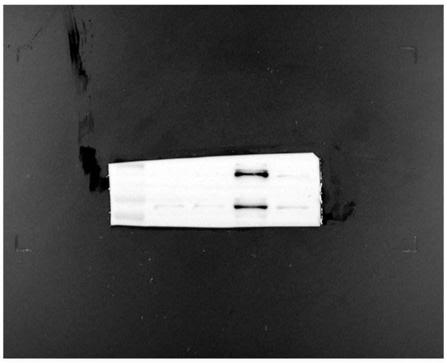

AMPKa

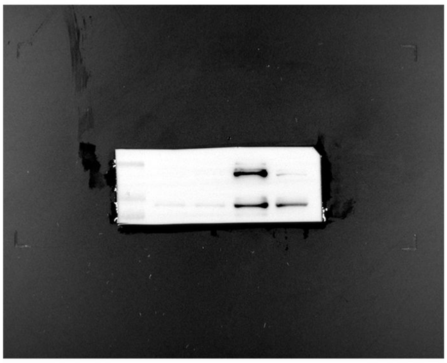

AMPKa

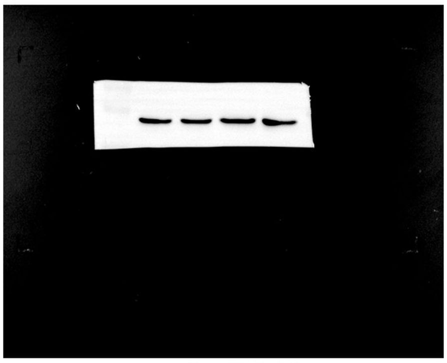

GAPDH

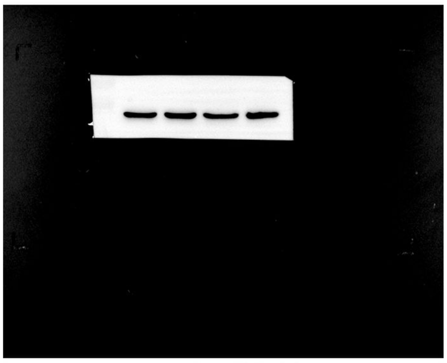

GAPDH

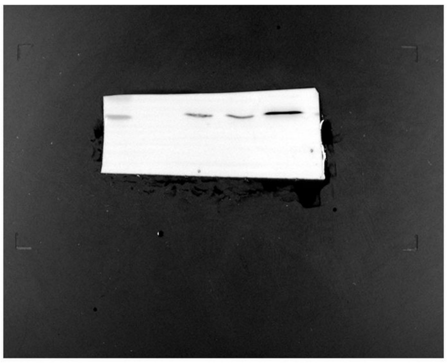
CC3

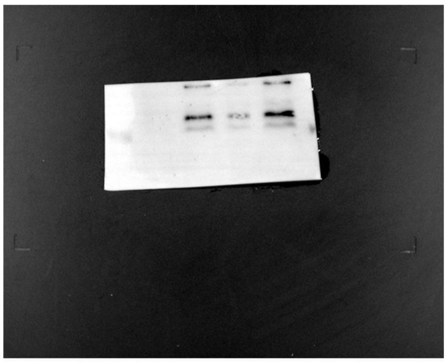
CC3

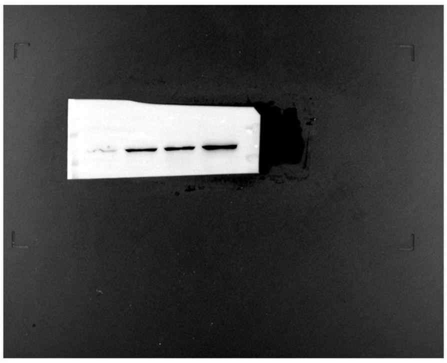
CP

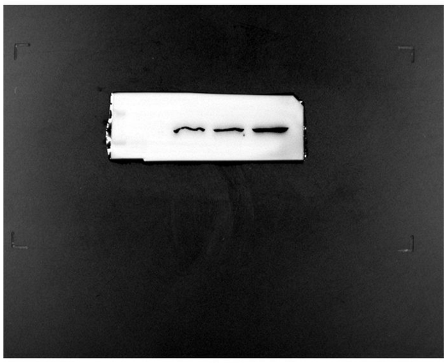
CP

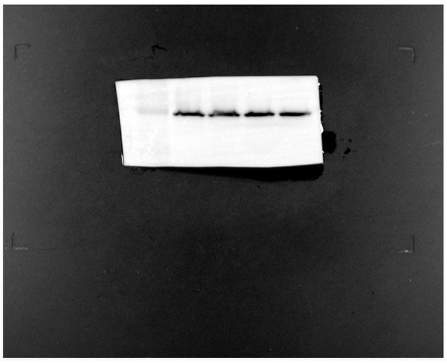
GAAPDH

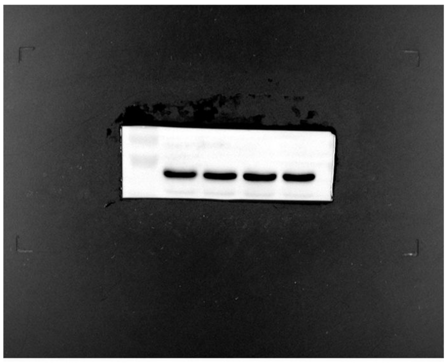
GAAPDH

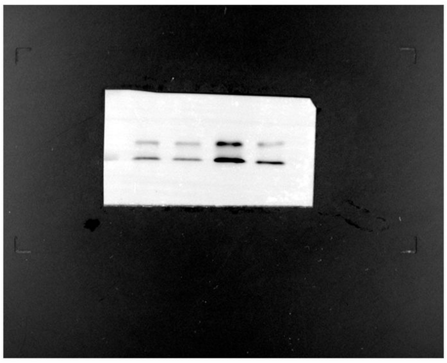
LC3

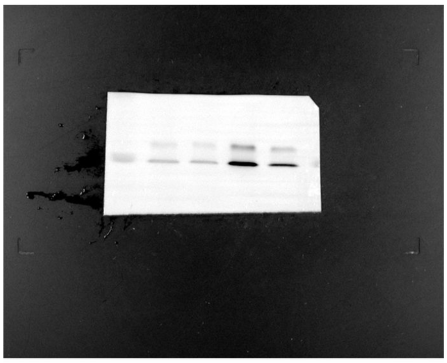
LC3

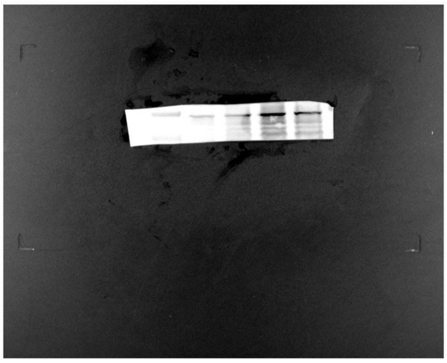
P-AMPKα

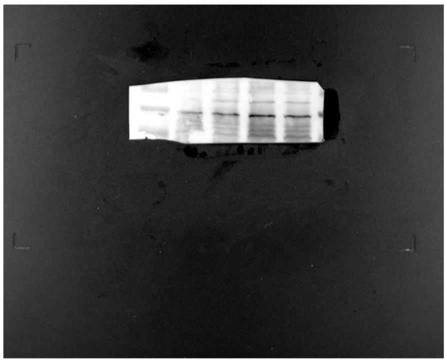
P-AMPKα

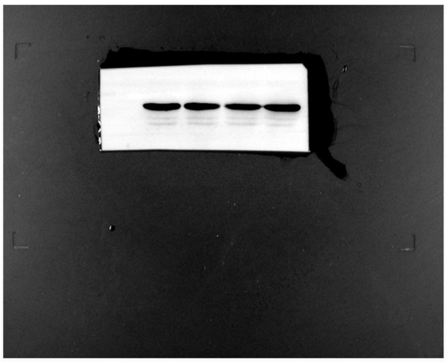

GAPDH

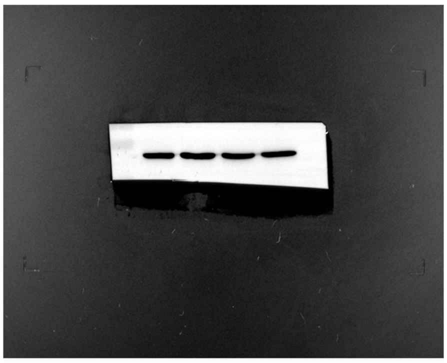

GAPDH

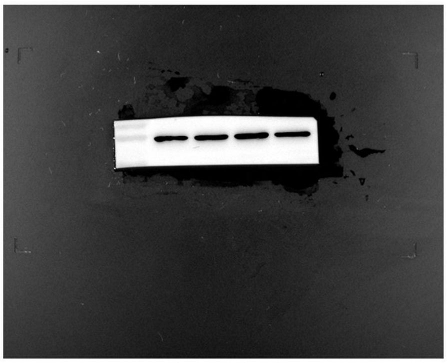

GAPDH

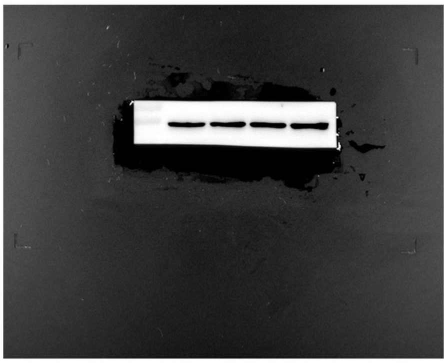

GAPDH

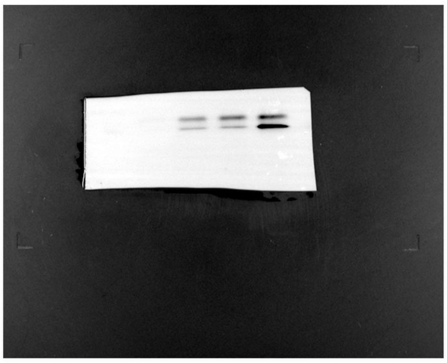

LC3

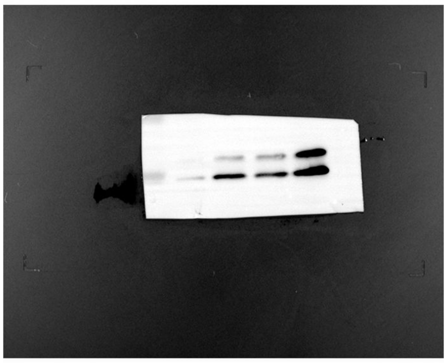

LC3

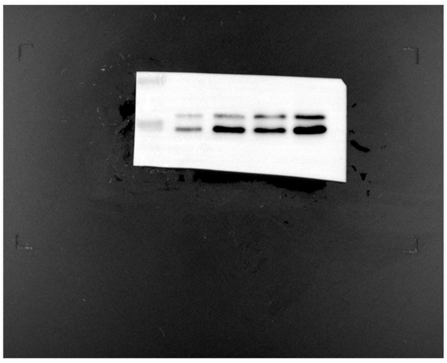

LC3

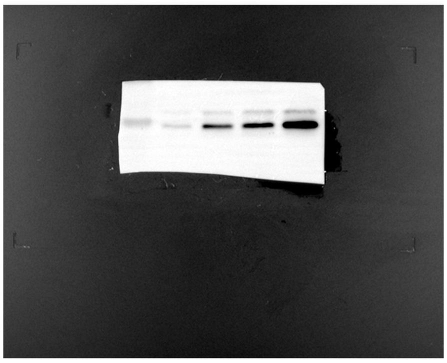

LC3

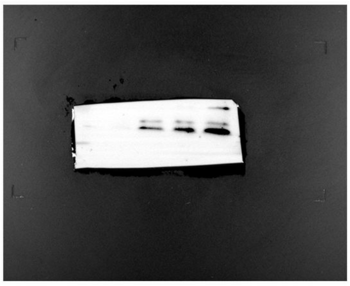
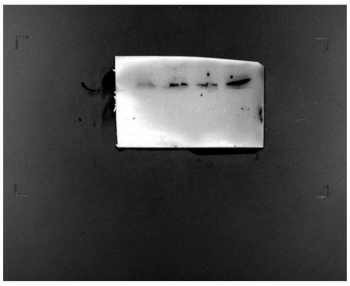
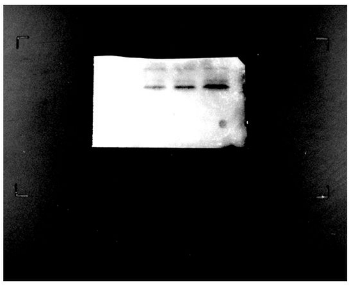
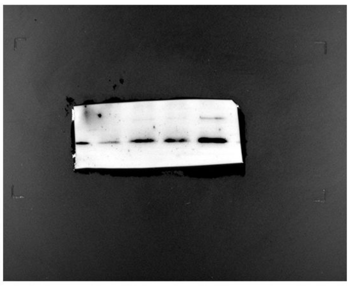
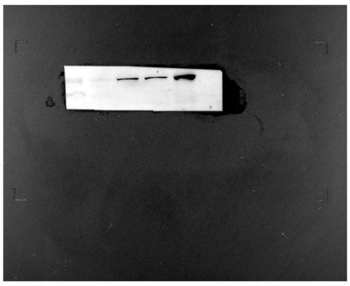
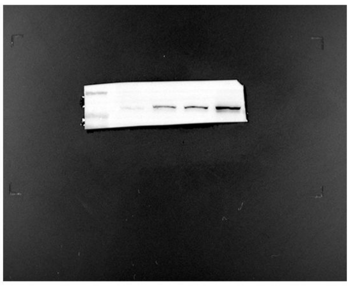
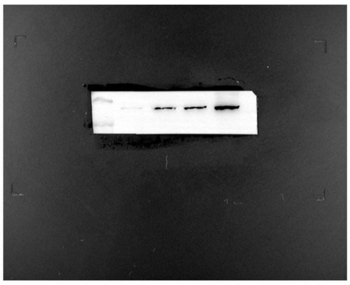
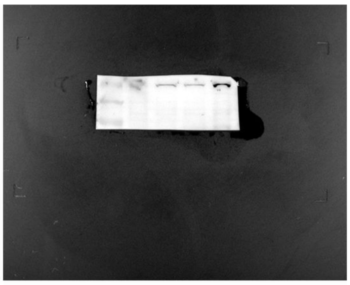
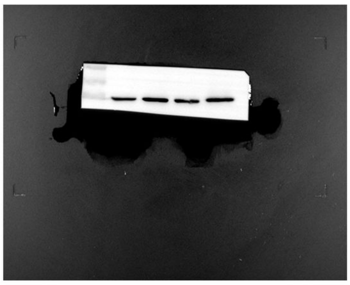
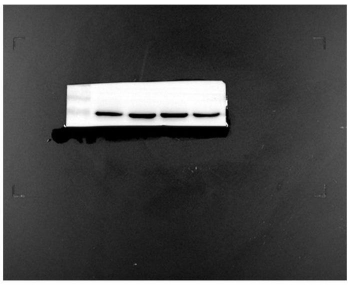
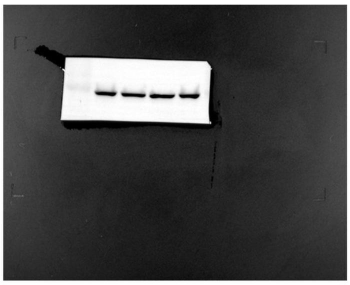
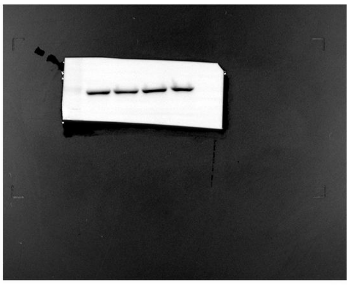

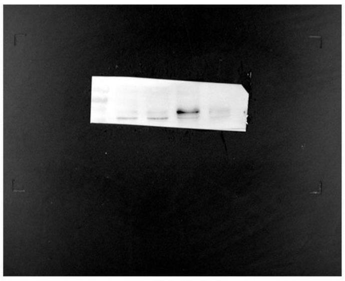

ATF4

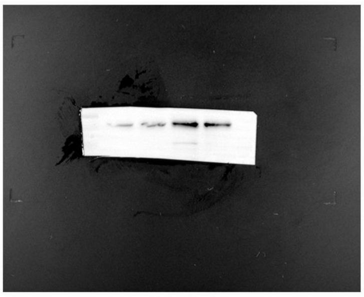

ATF4

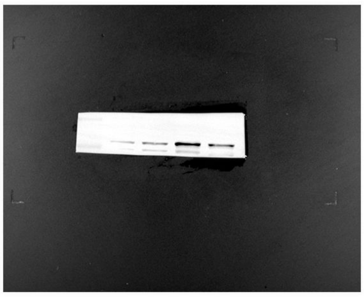

BIP

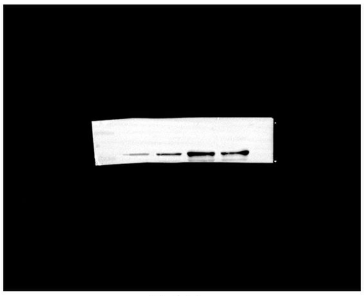

BIP

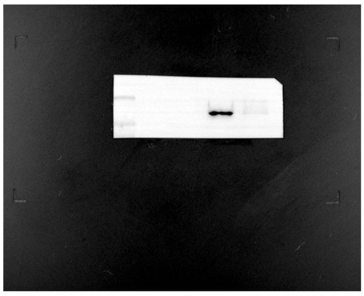

CP

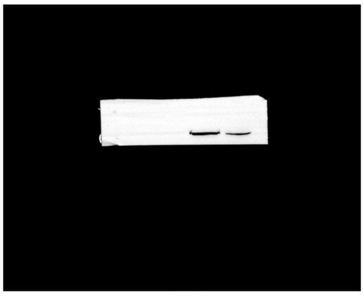

CP

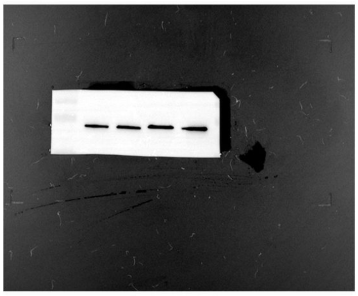

GAPDH

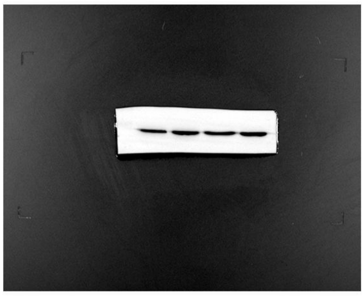

GAPDH

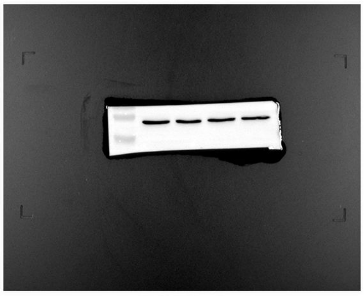

GAPDH

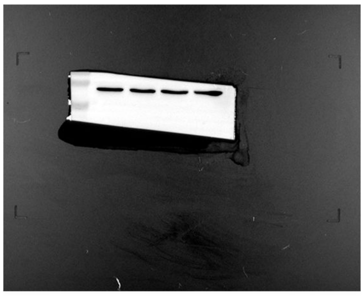

GAPDH

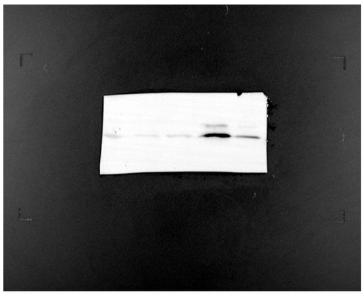

LC3

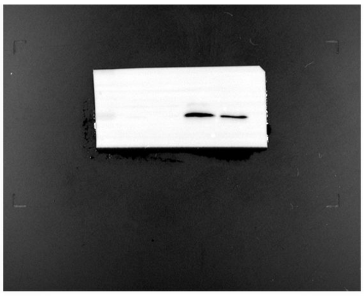

LC3

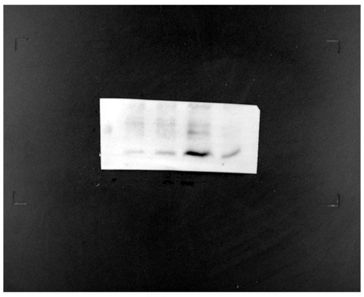

Noxa

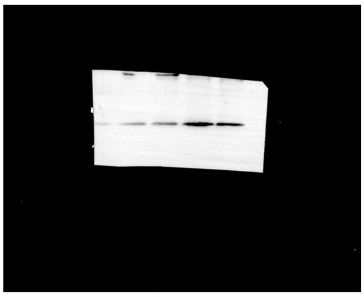

Noxa

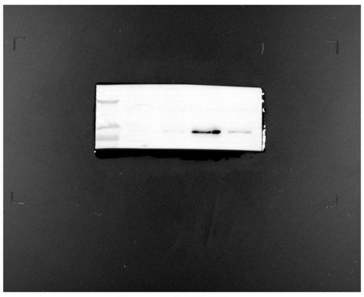

P-AMPKα

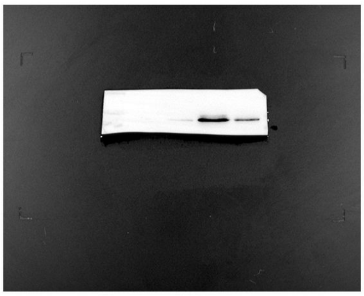

P-AMPKα

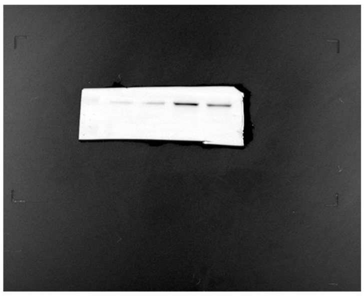

P-eif2α

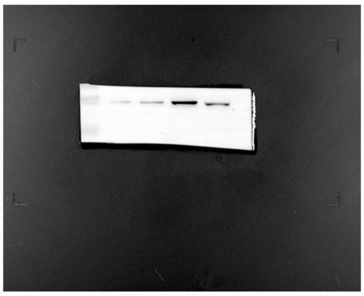

P-eif2α

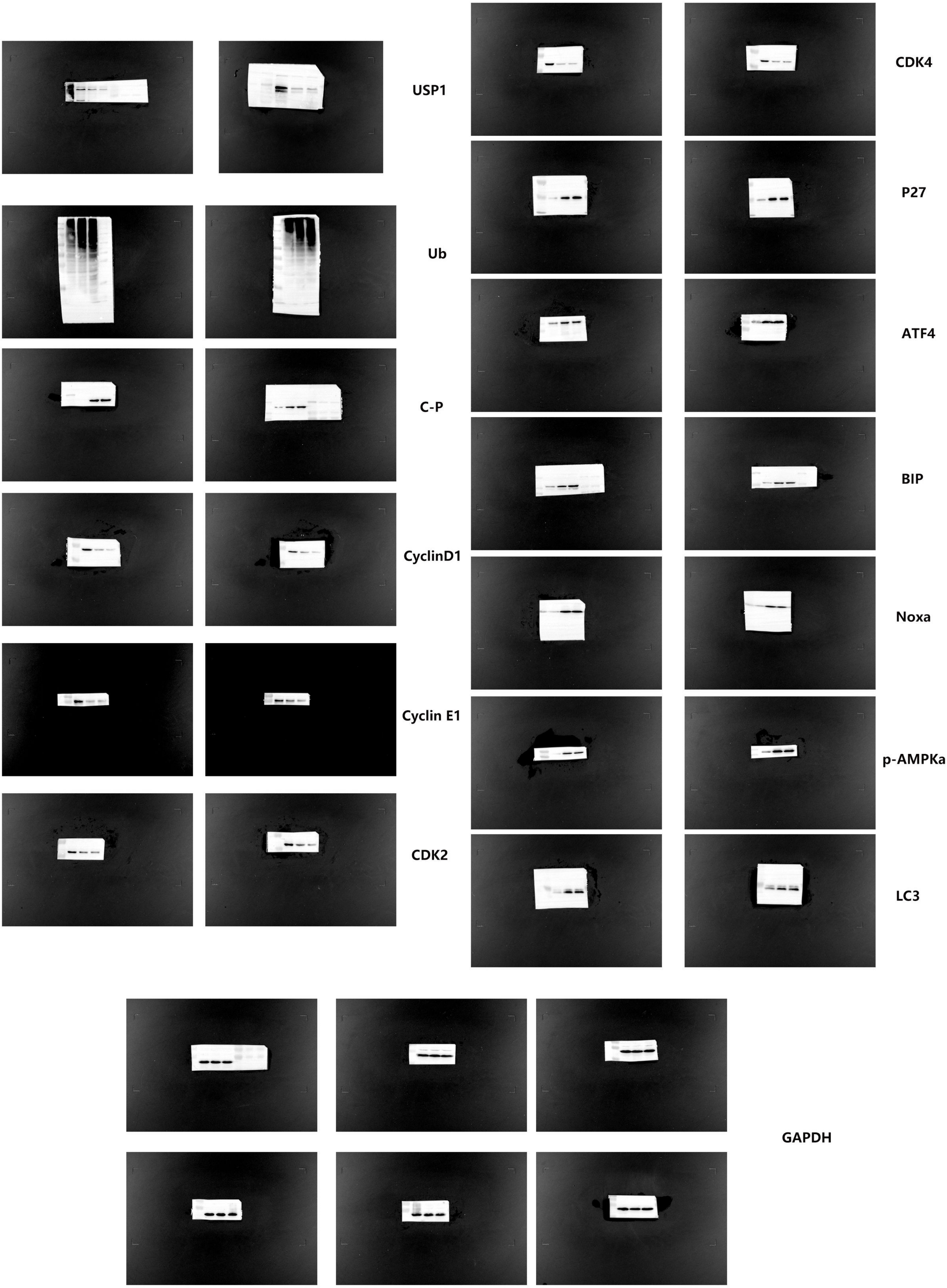
USP1
Ub
C-P
CyclinD1
Cyclin E1
CDK2
CDK4
P27
ATF4
BIP
Noxa
p-AMPKa
LC3
GAPDH
